# Design, Synthesis, and In Vitro Biological Activities of a Bio-Oxidizable Prodrug to Deliver Both ChEs and DYRK1A Inhibitors for AD Therapy

**DOI:** 10.3390/molecules24071264

**Published:** 2019-04-01

**Authors:** Anaïs Barré, Rabah Azzouz, Vincent Gembus, Cyril Papamicaël, Vincent Levacher

**Affiliations:** 1 VFP Therapies R&D; 1 rue Tesnière, 76130 Mont Saint-Aignan, France; anais.barre@insa-rouen.fr (A.B.); razzouz@vfp-therapies.com (R.A.); 2 Normandie University, UNIROUEN, INSA Rouen, CNRS, COBRA, 76000 Rouen, France; cyril.papamicael@insa-rouen.fr

**Keywords:** Alzheimer’s, acetylcholinesterase, butyrylcholinesterase, DYRK1A kinase, prodrug, INDY

## Abstract

Despite their side effects, cholinesterase (ChE) inhibitors remain the only approved drugs to treat Alzheimer’s disease patients, along with the *N*-methyl-d-aspartate (NMDA) receptor antagonist memantine. In the last few years, the dual-specificity tyrosine phosphorylation-regulated kinase 1A (DYRK1A) has also been studied as a promising target for the development of new drugs for this pathology. In this context, and based on our previous characterization of bio-oxidizable prodrugs of potent acetylcholinesterase (AChE) inhibitors, we envisioned a strategy involving the synthesis of a bio-oxidizable prodrug of both ChE and DYRK1A inhibitors. To this end, we fixed our interest on a known potent inhibitor of DYRK1A, namely INDY. The designed prodrug of both ChE and DYRK1A inhibitors was successfully synthesized, connecting both inhibitors by a carbonate link. This prodrug and its corresponding drug were then evaluated as ChEs and DYRK1A inhibitors. Remarkably, in vitro results were in accordance with the starting hypothesis, showing a relative inactivity of the prodrug against DYRK1A and ChEs and a potent inhibition of ChEs by the oxidized form. Molecular docking and kinetic studies of ChE inhibition by the active compound are also discussed in this report.

## 1. Introduction

**Scheme 1:**
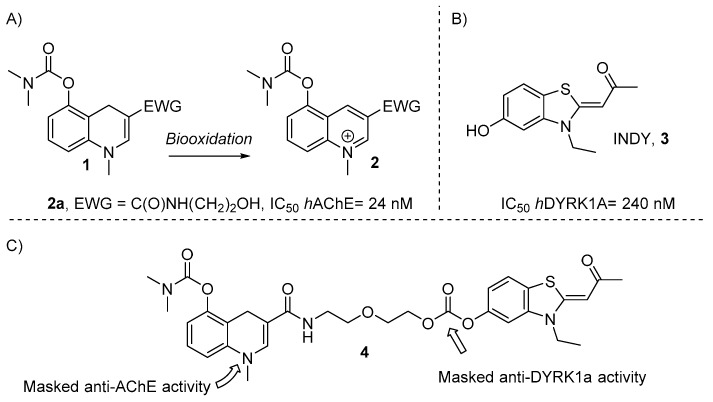
(**A**) Previous set of prodrugs **1** of potent AChE inhibitors **2** [41]. (**B**) Structure of the known DYRK1A inhibitor INDY **3** [43]. (**C**) This work: prodrug design to deliver AChE/DYRK1A inhibitors into the brain.

To date, Alzheimer’s disease (AD) represents 60 to 70 percent of dementia cases in the elderly, with a total 50 million cases in the world according World Health Organization data [1]. This mortal disease is still incurable, and the approved cholinesterase inhibitors (ChEIs) prescribed in the first stages of the disease, along with the *N*-methyl-d-aspartate (NMDA) receptor antagonist memantine, remain the only therapeutic tools for doctors to slow down the progression of memory and cognitive function impairments [2]. This is supported by the results of the many unsuccessfully clinical trials for other therapeutic approaches conducted in recent years [3,4]. Due to the multi-factorial aspect of AD and the observed synergic effects in several in vivo studies when two drugs are combined, researchers have turned their attention to this challenge and have developed hybrid compounds called multi-targeted directed ligands (MTDLs) [5,6,7,8,9]. Therefore, during the last decade, many active compounds based on the chemical structures of known AChEIs, and acting as AChEIs but also as inhibitors or ligands of other targets like BACE [10,11,12], monoamine oxidase [13,14], serotoninergic receptors [15,16,17], GS3K kinase [18,19], or others [20,21,22,23,24,25] have been described. Among other biological targets of interest for AD, the dual-specificity tyrosine phosphorylation-regulated kinase 1A (DYRK1A) is also considered to be very promising [26,27,28,29,30,31]. Overexpressed in postmortem human AD brain [32], this kinase is thought to be responsible for hyperphosphorylation of the Tau protein, leading to a decrease in its affinity for microtubules that causes a disorganization of the cytoskeleton responsible for neuronal apoptosis [33]. Other reports also suggest a role of the DYRK1A activity in the amyloid beta peptide (Aβ) accumulation [34,35]. For many years, our group has been interested in the development of new ChEIs, with the aim of replacing the marketed AChEIs by more specific and less toxic ones [36,37,38,39,40,41,42]. In this context, we previously patented and reported on the design of brain-penetrant bio-oxidative prodrugs of potent pseudo-irreversible AChE carbamate-based inhibitors (Scheme 1A) [41]. Interestingly, we showed that modulation at the C-3 position was feasible without detriment for the inhibitory activity against AChE, which thus constitutes a possible anchoring point for the design of new MTDL compounds. In particular, the highly potent compound **2a** (IC_50_ value of 24 nM), with an amino-PEG chain, was selected to address this issue. A survey of the literature also showed us that a potent and selective inhibitor of DYRK1A, namely INDY 3 [43], was easily accessible, and comprised a phenol function that could serve as an anchor to be attached to our AChEI (Scheme 1B). Furthermore, masking the phenol function could prevent any inhibitory activity against the kinase [43]. With these thoughts in mind, we envisioned the synthesis of compound **4**, designed to act as a dual prodrug of both AChE and DYRK1A inhibitors (Scheme 1C). The dihydroquinoline **4** should indeed be able to reach the brain to generate, after bio-oxidation, the corresponding quinonilium salt. The latter should exhibit an inhibitory effect not only against AChE, but also against DYRK1A, owing to the release of INDY **3** consecutively to the carbonate link hydrolysis. Here, we report the synthesis of compound **4** and the in vitro activity studies against ChEs and DYRK1A, as well as the molecular docking on ChEs.

## 2. Results and Discussion

### 2.1. Chemistry

**Scheme 2:**
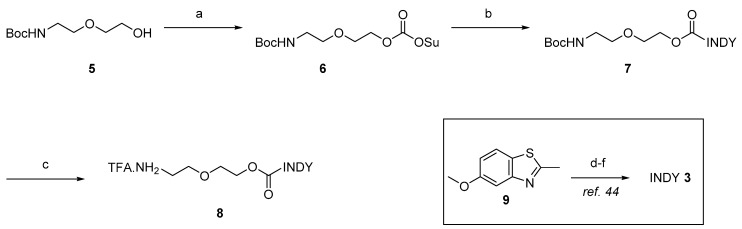
Preparation of INDY **3** and fragment **8**. *Reagents and conditions:* (**a**) DSC (2.0 equiv), NEt_3_ (2.0 equiv), ACN, rt, 2 h (quant.); (**b**) **3** (1.0 equiv), NaH (1.1 equiv) then, **6** (1.1 equiv), DMF, rt, 0.5 h (75%); (**c**) TFA excess, DCM, rt, 0.5 h (quant.); (**d**) Ethyl iodide (15 equiv), sealed tube, 80 °C, 5 days (96%); (**e**) Ac_2_O (2.3 equiv), NEt_3_ (2.4 equiv), ACN, reflux, 3 h (84%); (**f**) BBr_3_ (2.0 equiv), DCM, 0 °C to rt, 24 h (95%).

Starting from the commercially available Boc-protected amino-alcohol **5** (Scheme 2), the mixed succinimidyl carbonate **6** was obtained quantitatively by reaction with disuccinimidyl carbonate (DSC) in the presence of triethylamine at room temperature. This linker derivative **6** was then reacted with the sodium phenoxide of INDY **3**, initially formed by deprotonation of INDY **3** in presence of sodium hydride, to to furnish compound **7** in good yield (75%). Regarding the gram-scale synthesis of INDY **3**, it was prepared in three steps from the available benzothiazole **9** with a good overall yield (77%), and with only some minor experimental improvements of the procedure described by Hagiwara in 2004 [44]. Subsequently, the Boc group was classically removed in presence of an excess of trifluoroacetic acid (TFA) in dichloromethane at room temperature, leading to the expected ammonium salt **8** composed of the INDY moiety linked by a carbonate function to an amino-PEG chain.

**Scheme 3:**
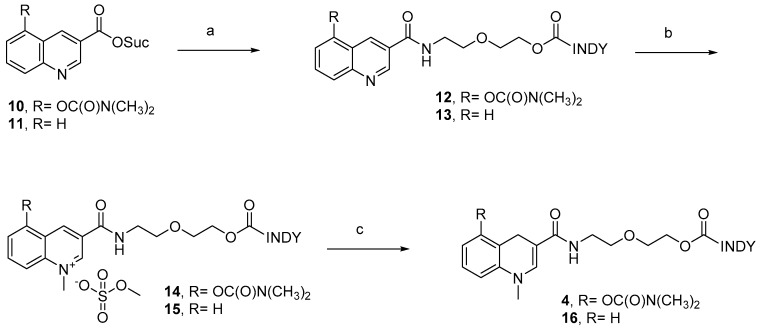
Grafting of fragment **8** to quinoline moieties. *Reagents and conditions:* (**a**) **8** (1.0 equiv), NEt_3_ (5.0 equiv), ACN, 0 °C to rt, 0.5 h (**12**: 71%, **13**: 98%); (**b**) Me_2_SO_4_ (1.1 equiv), DCE, sealed tube, 50 °C, 24 h (**14**: 93%, **15**: 86%); (**c**) BNAH (1.0 equiv), DCM, rt, 0.5 h (**4**: 72%, **16**: 80%).

Next, compound **8** was reacted with the activated succinimidyl ester **10** prepared according to our previously described synthesis [45], to afford product **12** at a 71% yield (Scheme 3). Under the same reaction conditions, with the succinimidyl ester **11** as reactant, compound **13** was also prepared, in order to demonstrate the influence of the carbamate at the C-5 position on the AChE inhibitory activity. The quinoline nitrogen of both compounds **12** and **13** was then selectively alkylated by action of dimethylsulfate to afford the corresponding quinolinium compounds **14** and **15** in excellent yields, 93% and 86%, respectively. As a last step to access the prodrugs, the selective 1,4-reduction of compounds **14** and **15** was performed with benzyl-1,4-dihydronicotinamide (BNAH) at room temperature in dichloromethane to afford the dihydroquinolines **4** and **16** in good yields.

### 2.2. In Vitro Activity Evaluation

Disposing of all envisioned products, the in vitro inhibitory activities on ChEs and DYRK1A were studied (Table 1). As expected, the bioprecursor **4** had only a modest effect on the inhibition of ChEs, with micromolar IC_50_ values. On the contrary, the oxidized form **14** displayed an excellent inhibition against *h*AChE, with a two-digit nanomolar IC_50_ value. Interestingly, the compound **14** also exhibited a good inhibitory activity against butyrylcholinesterase (BuChE), which could be an asset because this enzyme is also able to hydrolyze acetylcholine and, in contrast to AChE, it is not degraded during the progression of the AD [46,47]. Moreover, a better propidium iodide displacement inhibition was observed with compound **14** compared to donepezil, whereas the corresponding prodrug **4** did not seem to interact with the peripheral anionic site [48] (PAS), as indicated by a poor inhibition of 4% under the same conditions. It is worth noting that quinolinium salt **15**, lacking the carbamate moiety, did not show any significant inhibitory activity against either ChE, thus underlining the importance of this transferable group in the mechanism of ChE inhibition by this class of compounds. The same remark is true for the corresponding dihydroquinoline **16**. Unsurprisingly and as expected, none of the compounds showed any inhibitory activity against DYRK1A at one micromolar concentration compared to INDY.

**Figure 1:**
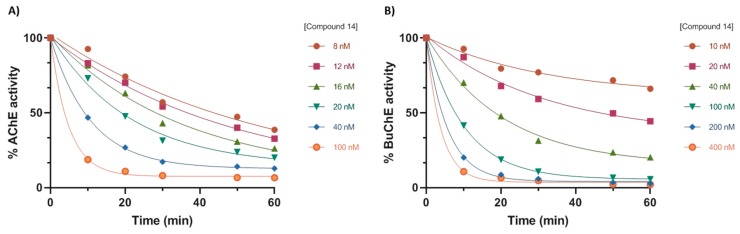
Plots of the decay curves for (**A**) *rh*AChE inhibition by compound **14** (8–100 nM) and (**B**) *h*BuChE inhibition by compound **14** (10–400 nM). This figure was made with Prism 8.0 [51].

To support the assumption of the pseudo-irreversible inhibition mechanism of both ChEs by compound **14**, kinetic studies were also performed using stopped time assays at different concentrations of ligand **14**. Confirming the carbamate-mediated type inhibition, a progressive inhibition over time was observed with both enzymes (Figure 1). Using a nonlinear regression fitting analysis, the pseudo-first-order inhibition rate constant (k_obs_) for each concentration of **14** was obtained and the equilibrium constant (Kc) and the carbamoylation rate constant (k_3_) was determined by the reported method [49,50]. Compound **14** showed good affinities for *h*AChE and *h*BChE, with Kc values of 229.8 and 659.1 nM respectively, and good rates of carbamate transfer (k_3_), with values of 0.408 and 0.574 min^−1^, respectively. Consequently, the second order rate constant Ki, defined as k_3_/Kc, and reflecting the carbamylation efficacy, was 1.78 μM^−1^·min^−1^ with *h*AChE and 0.87 μM^−1^·min^−1^ with *h*BuChE.

As a complement to the preceding studies, the ability of compounds **14** and **4** to cross the blood brain barrier (BBB) by passive diffusion was determined by PAMPA BBB assays. Unsurprisingly [39,40,41,42], the obtained values showed that the quinolinium salt **14** is not able to cross the BBB by this transport process (Pe = 0.00 × 10^−6^ cm·s^−1^), whereas the dihydroquinoline compound **4**, which is more lipophilic, diffuses moderately (Pe = 2.41 × 10^−6^ cm·s^−1^).

### 2.3. Docking

**Figure 2:**
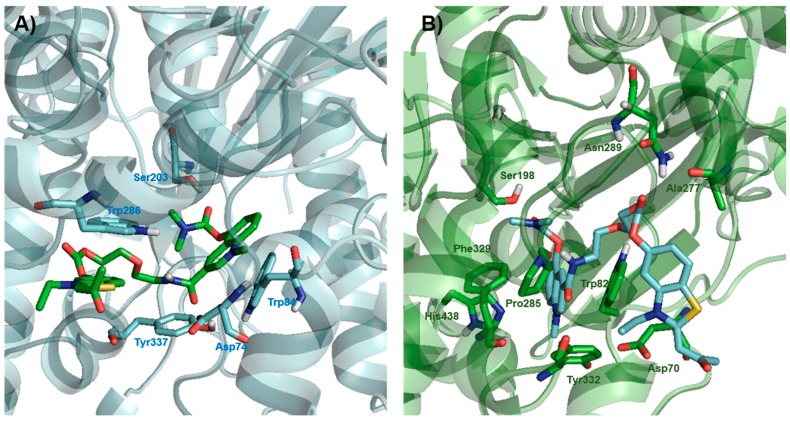
Top-scored pose obtained with Autodock Vina [52] for compound **14** positioned in (**A**) the active site of *rh*AChE (PDB ID: 4EY7), and (**B**) the active site of *h*BuChE (PDB ID: 1P0I). The compound and the selected side chains of the binding site residues are in stick and the protein in cartoon representation. This figure was made with PyMol [53].

In order to determine which favorable binding interactions can contribute to the potent inhibition of ChEs by compound **14**, docking experiments were performed. In the best docking pose on *h*AChE (PDB: 4EY7, Figure 2A), the carbamate group of compound **14** is favorably oriented toward the Ser 203 residue (1.96 Å). Whereas the quinolinium moiety has π−π interactions with Trp 86, the positive nitrogen resulted in a favorable attractive interaction with the Asp 74 residue (4.94 Å). Hydrogen bond interactions between the carbonyl of amide and Tyr 337 residue (2.7 Å), and between oxygen of the carbonate and Phe 295 residue (2.1 Å) also seem to be advantageous for the binding affinity. Supporting the potential interactions of compound **14** with the PAS, docking suggests that the benzothiazole moiety can be π-stacked with the Trp 286 residue (4.64 Å) of the PAS, with a possible π-sulfur interaction between the thiazole moiety of the ligand and the same residue (4.16 Å).

In the top-scored pose in *h*BuChE (PDB: 1P0I, Figure 2B), ligand **14** is favorably positioned in the catalytic site to permit the transfer of the carbamate group to Ser 198 (2.9 Å). The main interactions are illustrated by a π−π stacking between the quinolinum moiety of ligand **14** with the Trp 82 (4.1 Å), and by T-shaped stacking with the His438 and Phe329 residues (resp., 4.9 and 5.4 Å). An additional attractive charge interaction (4.7 Å) between the positive nitrogen and the Asp 70 residue could improve the binding affinity of compound **14** for *h*BuChE. Additionally, hydrogen bond interactions between the NH group of the amide function and the Pro285 residue (2.1 Å), as well as between the carbonyl of the carbonate group and the Asn 289 residue (2.2 Å), can also contribute to favorable binding interactions. According to this pose, the benzothiazole part of compound **14** would interact with Tyr 332 residue through a π−π interaction (T-stacking, 5.2 Å), and with Ala 277 through a π−alkyl interaction (5.1 Å).

## 3. Conclusions

In conclusion, the envisioned compound **4** and its corresponding quinolinium salt **14** were successfully prepared in six steps, starting from the previously described succinimidyl ester **10** and the Boc-protected amino-alcohol **5**. As expected, the quinolinium compound **14** showed a potent inhibitory activity against both the CAS and the PAS of *h*AChE and also, more pleasingly, against *h*BuChE. Put together, in vitro assay findings suggested that the observed dual inhibition of both AChE and BuChE by compound **14** could be explained by a pseudo-irreversible mechanism. This was also corroborated by the docking studies of **14** on both enzymes. Equally importantly, and as expected, we also showed that the carbonate link installed between INDY **3** and **2a** allowed transient masking of the inhibitory activity of INDY **3** against DYRK1A. Overall, these in vitro activity data are very encouraging for the future development of compound **4** as prodrug of selective central ChE and DYRK1A inhibitors. Further in vivo studies on small animals will be required to evaluate the potential of this prodrug approach and, in particular, to validate the release of INDY **3** into the brain.

## 4. Experimental Section

### 4.1. General Information

All purchased reagents were used as received. The solvents were used anhydrous directly from suppliers, or by drying with appropriate desiccants followed by distillation. Flash column chromatography was performed on silica gel (70–230 mesh), and thin layer chromatography used to monitor the reactions was carried out using commercial silica gel plates. NMR spectra (^1^H and ^13^C) were recorded using a Bruker Avance 300 MHz spectrometer ((Bruker, Wissembourg, France). Unless otherwise specified, DMSO-*d_6_* or CDCl_3_ served as internal standard (see Supplementary Materials). Peak multiplicities are reported as follow: s = singlet, d = doublet, t = triplet, q = quadruplet, dd = doublet of doublet, br = broad, apt = apparent triplet, and m = multiplet, coupling constants (*J* in Hz) and chemical shifts are given in ppm. High-resolution mass spectrometry (HRMS) was carried out on a Waters LCT XE spectrometer (WATERS, Guyancourt, France). Compounds **10** and **3** were synthesized using a previously described synthesis [44,45].

### 4.2. Chemistry

*tert-Butyl (2-(2-((((2,5-dioxopyrrolidin-1-yl)oxy)carbonyl)oxy)ethoxy)ethyl)carbamate* (**6**): Commercial *tert*-butyl *N*-[2-(2-hydroxyethoxy)ethyl]carbamate **5** (146 µL, 1.03 mmol, 1.0 equiv) was dissolved in dry acetonitrile (10 mL). Triethylamine (288 µL, 2.07 mmol, 2.0 equiv) and *N*,*N*′-disuccinimidyl carbonate (530 mg, 2.07 mmol, 2.0 equiv) were added, and the mixture was stirred for 2 h at room temperature. The solvent was removed in vacuo, and the residue was taken up in a saturated aqueous solution of sodium bicarbonate. The product was extracted three times with dichloromethane, dried over MgSO_4_, and concentrated to dryness. The crude product was purified by column chromatography (SiO_2_, EtOAc/PE = 1:2, *R_f_* = 0.37 [EtOAc/PE = 1:1]) to give the expected compound **6** (358 mg, quantitative yield) as a colorless oil. IR ν_max_/cm^−1^ 3387, 2977, 1737, 1702, 1220, 1201. ^1^H-NMR (300 MHz, CDCl_3_) **δ** 5.01 (br s, 1H, NH), 4.50–4.43 (m, 2H), 3.76–3.71 (m, 2H), 3.55 (t, *J* = 5.1 Hz, 2H), 3.32 (q, *J* = 5.2 Hz, 2H), 2.85 (s, 4H), 1.45 (s, 9H); ^13^C-NMR (75 MHz, CDCl_3_) **δ** 168.8, 155.9, 151.5, 78.9, 70.2, 69.8, 67.8, 40.1, 28.2, 25.3; HRMS (TOF-ESI^+^) calcd. for [M + H]^+^ C_14_H_23_N_2_O_8_ *m*/*z* 347.1449, found 347.1459.

*(Z)-tert-Butyl(2-(2-((((3-ethyl-2-(2-oxopropylidene)-2,3-dihydrobenzo[d]thiazol-5-yl)oxy)carbonyl)oxy)ethoxy)ethyl)carbamate* (**7**): INDY **3** (46 mg, 0.20 mmol, 1.0 equiv) and sodium hydride (60% dispersion in oil, 9 mg, 0.22 mmol, 1.1 equiv) were dissolved in dry dimethylformamide (4 mL). After 5 min, the succinymidyl carbonate **6** (74 mg, 0.22 mmol, 1.1 equiv) was added. After 20 min the reaction mixture was quenched with water at 0 °C, extracted with dichloromethane, washed with brine, dried over MgSO_4_, and evaporated to dryness. The crude product was purified by column chromatography (SiO_2_, EtOAc/PE = 1:2 to 1:1, *R_f_* = 0.65 [EtOAc 100%]) to afford the expected compound **7** (68 mg, 75%) as a white hygroscopic solid. IR ν_max_/cm^−1^ 2977, 1763, 1706, 1493, 1471, 1247, 1193. ^1^H-NMR (300 MHz, CDCl_3_) **δ** 7.47 (d, *J* = 8.4 Hz, 1H), 6.95–6.92 (m, 2H), 5.85 (s, 1H), 5.00 (s, 1H, NH), 4.38–4.35 (m, 2H), 3.97 (q, *J* = 7.2 Hz, 2H), 3.74–3.68 (m, 2H), 3.56–3.53 (m, 2H), 3.33–3.28 (m, 2H), 2.19 (s, 3H), 1.39 (s, 9H), 1.31 (t, *J* = 7.2 Hz, 3H); ^13^C-NMR (75 MHz, CDCl_3_) **δ** 191.5, 160.4, 156.0, 153.6, 150.2, 140.0, 124.8, 122.7, 115.5, 103.0, 90.5, 79.3, 70.3, 68.4, 67.8, 40.8, 40.3, 29.0, 28.4, 11.5; HRMS (TOF-ESI^+^) calcd. for [M + H]^+^ C_22_H_31_N_2_O_7_S *m*/*z* 467.1846, found 467.1850.

*(Z)-2-(2-((((3-Ethyl-2-(2-oxopropylidene)-2,3-dihydrobenzo[d]thiazol-5-yl)oxy)carbonyl)oxy)ethoxy)ethanaminium 2,2,2-trifluoroacetate* (**8**): Compound **7** (132 mg, 0.28 mmol, 1.0 equiv) was dissolved in dry dichloromethane (3 mL). Trifluoroacetic acid (0.65 mL, 8.49 mmol, 30 equiv) was added, and after 20 min the reaction mixture was evaporated to dryness to afford the expected compound **8** (quantitative yield) as a light brown oil. IR ν_max_/cm^−1^ 2925, 1764, 1673, 1180, 1131, 974, 798, 705. ^1^H-NMR (300 MHz, DMSO-*d*_6_) *δ* 7.80 (m, 4H), 7.41 (br s, 1H), 7.05 (d, *J* = 8.4 Hz, 1H), 6.15 (s, 1H), 4.41–4.35 (m, 2H), 4.17–4.09 (m, 2H), 3.79–3.73 (m, 2H), 3.65 (t, *J* = 5.2 Hz, 2H), 3.06–2.97 (m, 2H), 2.12 (s, 3H), 1.22 (t, *J* = 7.0 Hz, 3H); ^13^C-NMR (75 MHz, DMSO-*d*_6_) *δ* 190.2, 159.3, 153.3, 150.2, 140.1, 123.8, 123.1, 118.0, 115.8, 114.1, 104.3, 90.7, 68.1, 67.8, 66.8, 40.5, 38.6, 28.7, 11.5; HRMS (TOF-ESI^+^) calcd. for [M − CF_3_COO^−^]^+^ C_17_H_23_N_2_O_5_S *m*/*z* 367.1322, found 367.1331.

General procedure for synthesis of compounds **12** and **13**: Compound **8** (1.0 equiv) was dissolved in dry acetonitrile (0.1 M). The solution was cooled to 0 °C and triethylamine (5.0 equiv) was added. A solution of quinoline **10** (1.0 equiv) in dry acetonitrile (5 mL/mmol) was then added at room temperature. After 30 min, the reaction mixture was evaporated to dryness, the residue was taken up in water, extracted four times with dichloromethane, washed with brine, dried over MgSO_4_, and concentrated in vacuo. The crude product was purified by column chromatography on silica gel.

*(Z)-3-((2-(2-((((3-Ethyl-2-(2-oxopropylidene)-2,3-dihydrobenzo[d]thiazol-5-yl)oxy)carbonyl)oxy)ethoxy)ethyl)carbamoyl)quinolin-5-yl dimethylcarbamate* (**12**): Starting from compound **10** (174 mg, 0.487 mmol), compound **12** was obtained as a beige solid (214 mg, 71%) after purification by column chromatography (SiO_2_, EtOAc 100% to ACN/EtOAc = 4:1, *R_f_* = 0.18 [ACN/EtOAc = 1:3]). Mp 102–104 °C. IR ν_max_/cm^−1^ 2940, 1723, 1468, 1232, 1192, 969. ^1^H-NMR (300 MHz, CDCl_3_) *δ* 9.21 (d, *J* = 2.1 Hz, 1H), 8.75 (d, *J* = 2.0 Hz, 1H), 7.91 (d, *J* = 8.5 Hz, 1H), 7.72 (appt, *J* = 8.1 Hz, 1H), 7.41 (d, *J* = 8.4 Hz, 1H), 7.36 (d, *J* = 7.6 Hz, 1H), 7.02 (br s, 1H, NH), 6.89 (dd, *J* = 8.4, 2.0 Hz, 1H), 6.85 (d, *J* = 1.9 Hz, 1H), 5.84 (s, 1H), 4.48–4.41 (m, 2H), 3.90 (q, *J* = 7.3 Hz, 2H), 3.85–3.80 (m, 2H), 3.77 (br s, 4H), 3.25 (s, 3H), 3.05 (s, 3H), 2.23 (s, 3H), 1.28 (t, *J* = 7.2 Hz, 3H); ^13^C-NMR (75 MHz, CDCl_3_) *δ* 191.6, 165.8, 160.5, 154.5, 153.9, 150.1, 149.7, 148.2, 147.6, 140.0, 130.8, 130.6, 127.0, 126.6, 125.0, 122.8, 121.8, 119.7, 115.4, 102.9, 90.6, 69.7, 68.9, 67.7, 40.8, 40.1, 37.1, 36.9, 29.2, 11.6; HRMS (TOF-ESI^+^) calcd. for [M + H]^+^ C_30_H_33_N_4_O_8_S *m*/*z* 609.2014, found 609.2004.

*(Z)-3-Ethyl-2-(2-oxopropylidene)-2,3-dihydrobenzo[d]thiazol-5-yl (2-(2-(quinoline-3-carboxamido)ethoxy)ethyl) carbonate* (**13**): Starting from compound **10** (143 mg, 0.53 mmol), compound **13** was obtained as a white, very hygroscopic solid (270 mg, 98%) after purification by column chromatography (SiO_2_, EtOAc/PE = 1:1 to EtOAc 100%, *R_f_* = 0.17 [EtOAc 100%]). IR ν_max_/cm^−1^ 3237, 1753, 1649, 1246, 1067. ^1^H-NMR (300 MHz, CDCl_3_) **δ** 9.28 (d, *J* = 2.1 Hz, 1H), 8.56 (d, *J* = 1.7 Hz, 1H), 8.01 (d, *J* = 8.5 Hz, 1H), 7.73–7.63 (m, 2H), 7.44 (t, *J* = 7.5 Hz, 1H), 7.37 (br s, 1H, NH), 7.34 (d, *J* = 8.4 Hz, 1H), 6.85 (dd, *J* = 8.4, 1.9 Hz, 1H), 6.80 (d, *J* = 1.7 Hz, 1H), 5.79 (s, 1H), 4.48–4.38 (m, 2H), 3.88–3.48 (m, 8H), 2.20 (s, 3H), 1.22 (t, *J* = 7.1 Hz, 3H); ^13^C-NMR (75 MHz, CDCl_3_) **δ** 191.6, 165.9, 160.4, 153.8, 150.0, 149.0, 148.5, 139.9, 135.7, 131.2, 129.1, 128.7, 127.3, 126.8, 126.8, 124.8, 122.7, 115.4, 102.9, 90.5, 69.7, 68.7, 67.7, 40.7, 39.9, 29.1, 11.5; HRMS (TOF-ESI^+^) calcd. for [M + H]^+^ C_27_H_28_N_3_O_6_S *m*/*z* 522.1693, found 522.1710.

General procedure for synthesis of compounds **14** and **15**: Quinoline compound (1.0 equiv) was dissolved in dry dichloroethane (5 mL/mmol) in a sealed tube. Dimethyl sulfate (1.1 equiv) was then added, and the resulting solution was heated at 50 °C. After completion of the reaction, diethyl ether was added at room temperature, and the solid was filtered and dried in vacuo to afford the expected quinolinium compound **14**, which was used without further purification.

*(Z)-5-((Dimethylcarbamoyl)oxy)-3-((2-(2-((((3-ethyl-2-(2-oxopropylidene)-2,3-dihydrobenzo[d]thiazol-5-yl)oxy)carbonyl)oxy)ethoxy)ethyl)carbamoyl)-1-methylquinolin-1-ium methyl sulfate* (**14**): From quinoline **12** (133 mg, 0.219 mmol), compound **14** was isolated as a purple solid (150 mg, 93%). Mp 84–85 °C. IR ν_max_/cm^−1^ 3273, 3087, 2944, 1732, 1468, 1193, 1004, 740. ^1^H-NMR (300 MHz, DMSO-*d*_6_) *δ* 9.92 (s, 1H), 9.55–9.42 (m, 2H), 8.45–8.28 (m, 2H), 7.97–7.87 (m, 1H), 7.67 (d, *J* = 8.4 Hz, 1H), 7.34 (d, *J* = 1.9 Hz, 1H), 6.96 (dd, *J* = 8.4, 1.9 Hz, 1H), 6.13 (s, 1H), 4.67 (s, 3H), 4.42–4.30 (m, 2H), 4.14–4.05 (m, 2H), 3.82–3.73 (m, 2H), 3.73–3.64 (m, 2H), 3.64–3.54 (m, 2H), 3.36 (s, 3H), 3.26 (s, 3H), 3.02 (s, 3H), 2.12 (s, 3H), 1.19 (t, *J* = 7.0 Hz, 3H); ^13^C-NMR (75 MHz, DMSO-*d*_6_) *δ* 190.2, 162.4, 161.8, 158.9, 153.1, 153.0, 150.7, 150.0, 148.7, 139.9, 139.0, 138.5, 136.6, 127.5, 123.6, 123.0, 122.9, 116.2, 115.5, 104.0, 90.6, 68.6, 68.0, 67.8, 52.9, 46.1, 44.1, 40.3, 39.7, 36.8, 36.6, 28.8, 11.4; HRMS (TOF-ESI^+^) calcd. for [M − MeSO_4_^−^]^+^ C_31_H_35_N_4_O_8_S *m*/*z* 623.2170, found 623.2170.

*(Z)-3-((2-(2-((((3-Ethyl-2-(2-oxopropylidene)-2,3-dihydrobenzo[d]thiazol-5-yl)oxy)carbonyl)oxy)ethoxy)ethyl)carbamoyl)-1-methylquinolin-1-ium methyl sulfate* (**15**): From quinoline **13** (75 mg, 0.144 mmol), compound **15** was isolated as a pale pink solid (80 mg, 86%). Mp 183 °C (decomp.). IR ν_max_/cm^−1^ 3308, 3067, 1755, 1463, 1230, 974, 745. ^1^H-NMR (300 MHz, DMSO-*d*_6_) *δ* 9.88 (s, 1H), 9.63 (s, 1H), 9.28 (br t, *J* = 5.5 Hz, 1H), 8.52 (d, *J* = 8.9 Hz, 1H), 8.47 (d, *J* = 8.1 Hz, 1H), 8.37–8.28 (m, 1H), 8.12–8.03 (m, 1H), 7.70 (d, *J* = 8.4 Hz, 1H), 7.35 (d, *J* = 1.8 Hz, 1H), 6.98 (dd, *J* = 8.4, 2.0 Hz, 1H), 6.13 (s, 1H), 4.66 (s, 3H), 4.41–4.34 (m, 2H), 4.09 (q, *J* = 7.1 Hz, 2H), 3.81–3.73 (m, 2H), 3.73–3.65 (m, 2H), 3.63–3.55 (m, 2H), 3.36 (s, 3H), 2.12 (s, 3H), 1.19 (t, *J* = 7.0 Hz, 3H); ^13^C-NMR (75 MHz, DMSO-*d*_6_) *δ* 190.1, 161.9, 158.9, 153.1, 150.0, 149.8, 144.9, 139.8, 138.7, 136.7, 131.3, 130.6, 128.1, 127.6, 123.6, 122.9, 119.3, 115.5, 104.0, 90.5, 68.6, 67.9, 67.8, 52.8, 45.6, 40.3, 39.6, 28.8, 11.4; HRMS (TOF-ESI^+^) calcd. for [M − MeSO_4_^−^]^+^ C_28_H_30_N_3_O_6_S *m*/*z* 539.1848, found 536.1850.

General procedure for synthesis of compounds 4 and **16**: Quinolinium salt (1.1 equiv) and BNAH (1.0 equiv) were dissolved in degassed dichloromethane (10 mL/mmol) in the dark, under argon atmosphere. After 30 min of stirring at room temperature in the dark, the solution was washed three times with degassed water, dried over MgSO_4_, and evaporated to dryness to give the expected dihydroquinoline, which was used without further purification.

*(Z)-3-((2-(2-((((3-Ethyl-2-(2-oxopropylidene)-2,3-dihydrobenzo[d]thiazol-5-yl)oxy)carbonyl)oxy)ethoxy)ethyl)carbamoyl)-1-methyl-1,4-dihydroquinolin-5-yl dimethylcarbamate* (**4**): From quinolinium salt **14** (113 mg, 0.154 mmol) and BNAH (30 mg, 140 mmol), the expected dihydroquinoline **4** (63 mg, 72%) was obtained as a pale orange solid. Mp 89–91 °C. IR ν_max_/cm^−1^ 2922, 1717, 1667, 1469, 1231. ^1^H-NMR (300 MHz, DMSO-*d*_6_) *δ* 7.73 (d, *J* = 8.4 Hz, 1H), 7.46–7.39 (m, 2H), 7.15–7.08 (m, 2H), 7.05 (dd, *J* = 8.4, 1.9 Hz, 1H), 6.64 (d, *J* = 8.2 Hz, 2H), 6.14 (s, 1H), 4.38–4.31 (m, 2H), 4.12 (q, *J* = 6.9 Hz, 2H), 3.73–3.67 (m, 2H), 3.51 (appt, *J* = 6.0 Hz, 2H), 3.44 (s, 2H), 3.34–3.27 (m, 2H), 3.14 (s, 3H), 3.06 (s, 3H), 2.91 (s, 3H), 2.12 (s, 3H), 1.20 (t, *J* = 7.1 Hz, 3H); ^13^C-NMR (75 MHz, DMSO-*d*_6_) *δ* 190.1, 166.8, 159.0, 153.6, 153.2, 150.1, 149.8, 140.5, 140.0, 138.5, 127.3, 123.6, 122.9, 116.0, 115.7, 115.3, 109.4, 104.2, 99.6, 90.5, 69.3, 67.9, 40.3, 38.8, 38,6, 36.4, 36.2, 28.8, 21.1, 11.5; HRMS (TOF-ESI^+^) calcd. for [M + H]^+^ C_31_H_37_N_4_O_8_S *m*/*z* 625.2327, found 625.2328.

*(Z)-3-Ethyl-2-(2-oxopropylidene)-2,3-dihydrobenzo[d]thiazol-5-yl (2-(2-(1-methyl-1,4-dihydroquinoline-3-carboxamido)ethoxy)ethyl) carbonate* (**16**): From quinolinium salt **15** (61 mg, 0.094 mmol) and BNAH (18 mg, 0.084 mmol), the expected dihydroquinoline **16** (36 mg, 80%) was obtained as a pale orange solid. ^1^H-NMR (300 MHz, CDCl_3_) *δ* 7.44 (d, *J* = 8.3 Hz, 1H), 7.16 (s, 1H), 7.07 (appt, *J* = 7.6 Hz, 1H), 7.01–6.86 (m, 3H), 6.80 (appt, *J* = 7.3 Hz, 1H), 6.65 (d, *J* = 8.1 Hz, 1H), 5.86 (s, 1H), 5.79 (br s, 1H, NH), 4.50–4.39 (m, 2H), 3.94 (q, *J* = 7.2 Hz, 2H), 3.83–3.76 (m, 2H), 3.73 (s, 2H), 3.70–3.63 (m, 2H), 3.63–3.54 (m, 2H), 3.13 (s, 3H), 2.24 (s, 3H), 1.32 (t, *J* = 7.1 Hz, 3H); ^13^C-NMR (75 MHz, CDCl_3_) *δ* 191.6, 167.7, 160.5, 153.9, 150.2, 140.4, 140.1, 139.2, 129.6, 127.5, 125.0, 122.9, 122.5, 121.7, 115.5, 112.5, 102.9, 98.9, 90.6, 70.3, 68.6, 67.8, 40.9, 39.3, 38.7, 29.2, 26.5, 11.6; HRMS (TOF-ESI^+^) calcd. for [M + H]^+^ C_28_H_32_N_3_O_6_S *m*/*z* 538.2006, found 536.2001.

### 4.3. Biological Evaluation

#### 4.3.1. *h*AChE and *h*BuChE Inhibition Assays

Inhibitory capacity of compounds on *h*AChE and *h*BuChE biological activity was evaluated through the use of Ellman’s spectrometric method [54]. Acetylthiocholine iodide (ATCI), butyrylcholine iodide (BTCI) and 5,5-dithiobis-(2-nitrobenzoic) acid (DTNB) were purchased from Sigma Aldrich. Lyophilized *h*AChE and *h*BuChE were purchased from Aldrich, and were diluted in phosphate buffer (pH 8) so as to give an enzyme solution with 0.25 units/mL enzyme activity. In the procedure, 100 μL of 0.3 mM DTNB dissolved in phosphate buffer pH 7.4 were added into the 96 well plate, followed by 50 μL of the tested compound solution and 50 μL of enzyme solution. After 5 min of preincubation, the reaction was then initiated by the injection of 50 μL of 10 mM ATCI or BTCI solution. The activity was determined by measuring the increase in absorbance at a wavelength of 412 nm every minute for 10 min using a 96 well microplate reader (Varian Carry^®^ 50 UV-Vis spectrophotometer, Darmstadt, Germany). Tested compounds were initially dissolved in analytical grade DMSO. Donepezil or Tacrine was used as reference standard. All assays were performed in duplicate, and IC_50_ values were determined graphically from 8 points inhibition curves using the Prism^®^ software [51].

For the time-dependent experiments, assays were performed using a 96 well microplate reader to a final volume of 250 μL, a final enzyme concentration of 0.05 U·mL^−1^ and an ATCI (or BTCI) and DNTB concentrations of 0.3 and 0.5 mM respectively in phosphate buffer (pH 8). At the starting time (t0), the inhibitor at various concentration (150 μL) was added to an equivalent volume of *h*AChE (or *h*BuChE). The mixture was then incubated at 37 °C and, at different time points, 50 μL aliquots were transferred to wells containing a solution of ATCI (or BTCI) and DTNB in phosphate buffer (200 μL) and the activity was determined by measuring the changes in absorbance at 412 nm every minute for 10 min. Blank experiments were also performed without inhibitor. The first order inhibition constant k_obs_ was determined for all inhibitor concentrations using a nonlinear regression. Data were fitted with the equation, A = A_0_ exp(−k_obs_·t) + A_∞_ with A, A_0_, A_∞_ being ratios of the inhibited enzyme activity to the control activity at time t, 0, and ∞. Double reciprocal plots of k_obs_ versus the inhibitor concentration were made to determine the equilibrium constant Kc and the carbamoylation rate constant k_3_, following the described method [50].

#### 4.3.2. Propidium Competition Assays

The affinity of compound **14** for the peripheral binding site of *ee*AChE was tested using a propidium iodide displacement assay. The assays were performed at the Centre d’Etudes et de Recherche sur le Médicament de Normandie (CERMN) in Caen, France. An increase in fluorescence on complexation of propidium iodide in *ee*AChE peripheral site was observed. Fluorescence intensity was monitored using a microplate reader. Fluorescence measurements were performed in 96 well plates, with a final volume in each well of 200 μL. A solution of a mixture of *ee*AChE (5 U from Aldrich) and compound **14** (or donepezil as a control) (10^−5^ M) in Tris/HCl buffer (1 mM, pH 8) (150 μL) was incubated for 6 h at 25 °C. A solution of propidium iodide in the same buffer (1 μM, 50 μL) was then added to the each well. After 10 min, the fluorescence measurement was performed (excitation wavelength of 535 nm, emission wavelength of 595 nm). Each assay was performed in triplicate.

#### 4.3.3. DYRK1A Inhibition Assay

Evaluation of the effects of compounds on the activity of the human recombinant DYRK1A was made using the LANCE^®^ detection method, and was quantified by measuring the phosphorylation of Ulight-CFFKNIVTPRTPPPSQGK-amide (MBP). The assays were conducted by Eurofins Pharma Discovery in Bois l’Evêque, France. The test compounds (or staurosporine as reference or water as control) were mixed with the enzyme (11.2 ng) in a pH 7.4 Hepes/Tris buffer (40 mM Hepes/Tris, 0.8 mM EGTA/Tris, 8 mM MgCl_2_, 1.6 mM DTT, 0.008% Tween 20). Next, 100 nM of Ulight-CFFKNIVTPRTPPPSQGK-amide (MBP) and 10 µM ATP were added to initiate the reaction, and the mixture was incubated for 30 min at 25 °C. A blank without enzyme was also performed for control basal measurements. The experiment was stopped by addition of 13 mM EDTA, followed by the addition of the anti-phospho-MBP antibody labeled with europium chelate after 5 min. After 1 h of incubation, the fluorescence was measured (excitation wavelength of 337 nm, emission wavelength of 620 and 665 nm). The enzyme activity corresponds to the ratio of signal measured at 665 nm to the signal measured at 620 nm. Outputs are expressed as a percentage of inhibition of the control DYRK1A activity.

#### 4.3.4. PAMPA-BBB Permeability Assay

Assays were performed at the Centre d’Etudes et de Recherche sur le Médicament de Normandie (CERMN) in Caen, France. These assays were realized according to the methodology developed by PION, by means of the Pampa-BBB Explorer™ system. This system allows the measurement of the crossing velocity of a compound from one compartment to another through an artificial membrane at pH = 7.4. The experiment was replicated 6 times in 4 h, with quantification by UV-spectra reading. The result is given in Pe [cm·s^−1^], and an interpretation by comparison with standard compounds is proposed. The studied compounds were diluted at 20 mM in DMSO, then diluted at 100 µL in Prisma HT Buffer pH 7.4 (pION). 200 µL of this solution was placed in the wells of the donor plate. 5 μL of BBB-1 Lipid was placed in the filters of the acceptor plate. 200 µL of Brain Sink Buffer was added in the wells of the acceptor plate. The sandwich was then assembled and incubated for 4 h at room temperature, without stirring. The sandwich was then separated and the UV-vis spectra of the donor and acceptor compartments were determined with the plate reader (Tecan infinite M200, Männedorf, Switzerland). Pe were calculated with the PAMPA Explorer software v.3.7 (pION Inc, Billerica, MA, USA). Standard compounds used were corticosterone and theophylline.

### 4.4. Docking Simulation

The crystal structures (PDB ID: 4EY7 and PDB 1P0I) of *h*ACHE in complex with donepezil and of *h*BuChE in complex with butyrate were downloaded from the RCSB Web site (http://www.pdb.org). The ligands were drawn in Marvin Sketch [55], hydrogen was added and their energies were minimized with Chimera 1.5.3 software with default setting [56]. Autodock Tools (ADT 1.5.6) was used to add Gasteiger charges to the ligand and to control the flexible torsions [57]. Before the docking, the protein was prepared with the DockPrep module of Chimera 1.5.3 software (National Institute of Health, Bethesda, MD, USA) using the default setting. The original crystal ligand and water molecules were removed from the protein−ligand complexes, and ADT 1.5.6 was used with the default setting to add polar hydrogen atoms and to assign Kollman charges. The docking studies of compound **14** with the *h*AChE and *h*BuChE proteins were carried out using AutodockVina [52] with the docking parameters set at default values. For *rh*AChE, the box center was set to −13.8673/−48.5081/34.708, and the box size was set to 25/32/27 Å. For *h*BuChE, the box center was set to 139.328/112.881/37.9116; the box size was set to 35/35/35 Å and catalytic triad and Trp82 were selected as flexible residues. Visualization was performed in PyMol 0.99rc6 [53].

## Figures and Tables

**Table 1:** In vitro evaluation of compounds **4**, **14**–**16**.

Compound	IC_50_ *h*AChE (nM) ^1^	IC_50_ *h*BuChE (nM) ^1^	DYRK1a Inhibition (%) ^1,2^	Propidium Iodide Displacement Inhibition (%) ^3^
**2a** ^2^	37.1 ± 0.1	1440.2 ± 286	0	-
**14**	81.4 ± 0.4	482 ± 21	5	26 ± 1
**15**	>10,000	6400 ± 714	6	-
**4**	1023.1 ± 72.1	961 ± 22	8	4 ± 2
**16**	>10,000	>10,000	7	-
Indy	>10,000	>10,000	92	-
Donepezil	15.3 ± 1.7	-	-	18 ± 1
Tacrine	-	17.1 ± 0.1	-	-

^1^ Mean value of duplicate. ^2^ Percentage of DYRK1A inhibition at 1 μM (10 μM ATP concentration). ^3^ Mean value of triplicate.

## Supplementary Materials

The following are available online, Copies of ^1^H and ^13^C-NMR spectra for all synthesized compounds are provided.
