# Supplementary material for: Design, Synthesis, and In Vitro Biological Activities of a Bio-Oxidizable Prodrug to Deliver Both ChEs and DYRK1A Inhibitors for AD Therapy
Source: Molecules. 2019 Apr 1;24(7):1264. doi: 10.3390/molecules24071264 (PMC6479981; doi:10.3390/molecules24071264)

SUPPORTING MATERIAL

Design, Synthesis and In Vitro Biological Activities of a Bio-oxidizable Prodrug to Deliver both ChEs and DYRK1A Inhibitors for AD Therapy

Anaïs Barré [1,2], Rabah Azzouz[1], Vincent Gembus [1,*] Cyril Papamicaël [2] and Vincent Levacher [2,*]

[1] VFP Therapies R&D; 1 rue Tesnière, 76130 Mont Saint-Aignan, France
[2] Normandie Univ, UNIROUEN, INSA Rouen, CNRS, COBRA, 76000 Rouen, France

* Correspondence: vincent.levacher@insa-rouen (V.L); vgembus@vfp-therapies.com (V.G.)

Content:

- NMR spectra (^{1}H/DEPT 135/^{13}C) of compounds **6**, **7**, **8**, **9**, **12**, **13**, **14**, **4**, **15** and **16**

- ^{1}H NMR spectrum in $CDCl_3$ of compound (**6**)

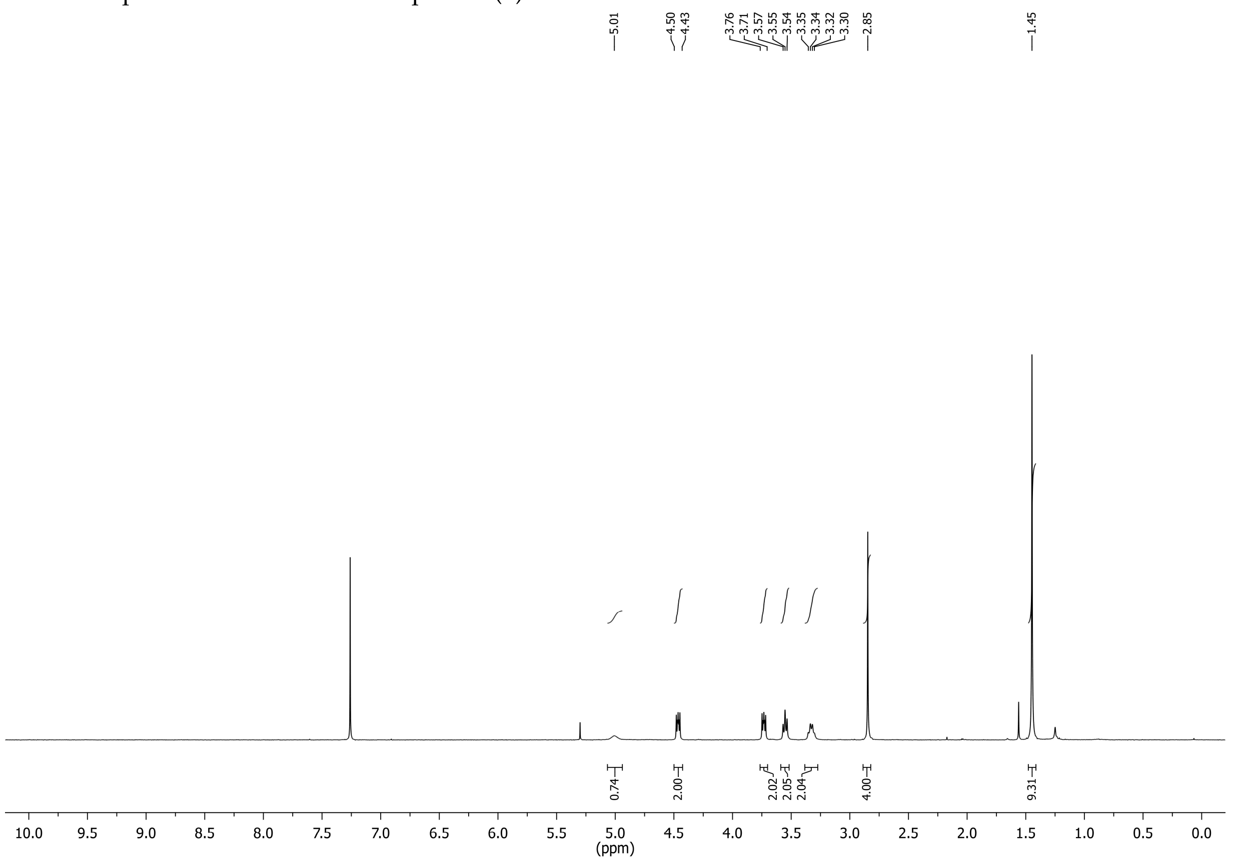

- DEPT-135 NMR spectrum in $CDCl_3$ of compound (**6**)

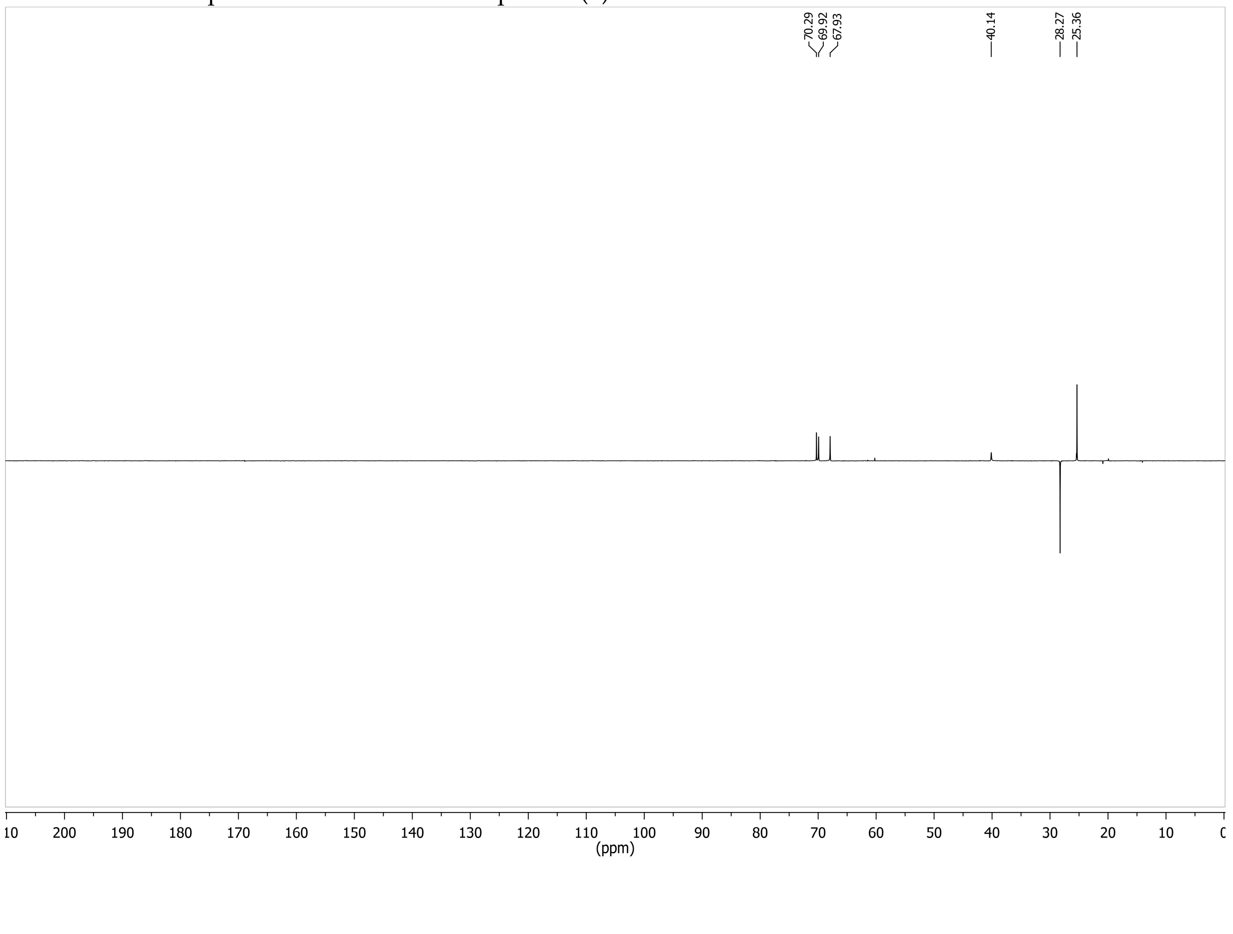

- ^{13}C NMR spectrum in $CDCl_3$ of compound (**6**)

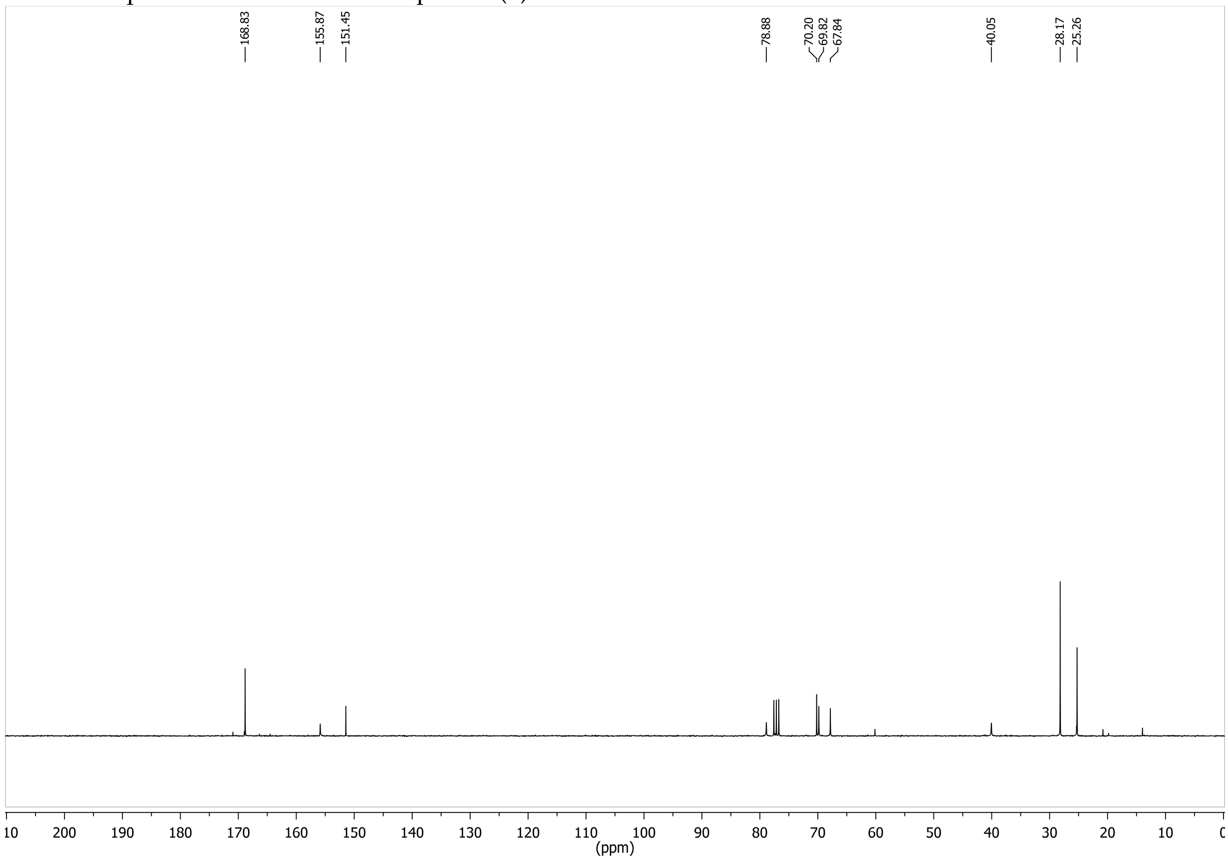

- ^{1}H NMR spectrum in $CDCl_3$ of compound (**7**)

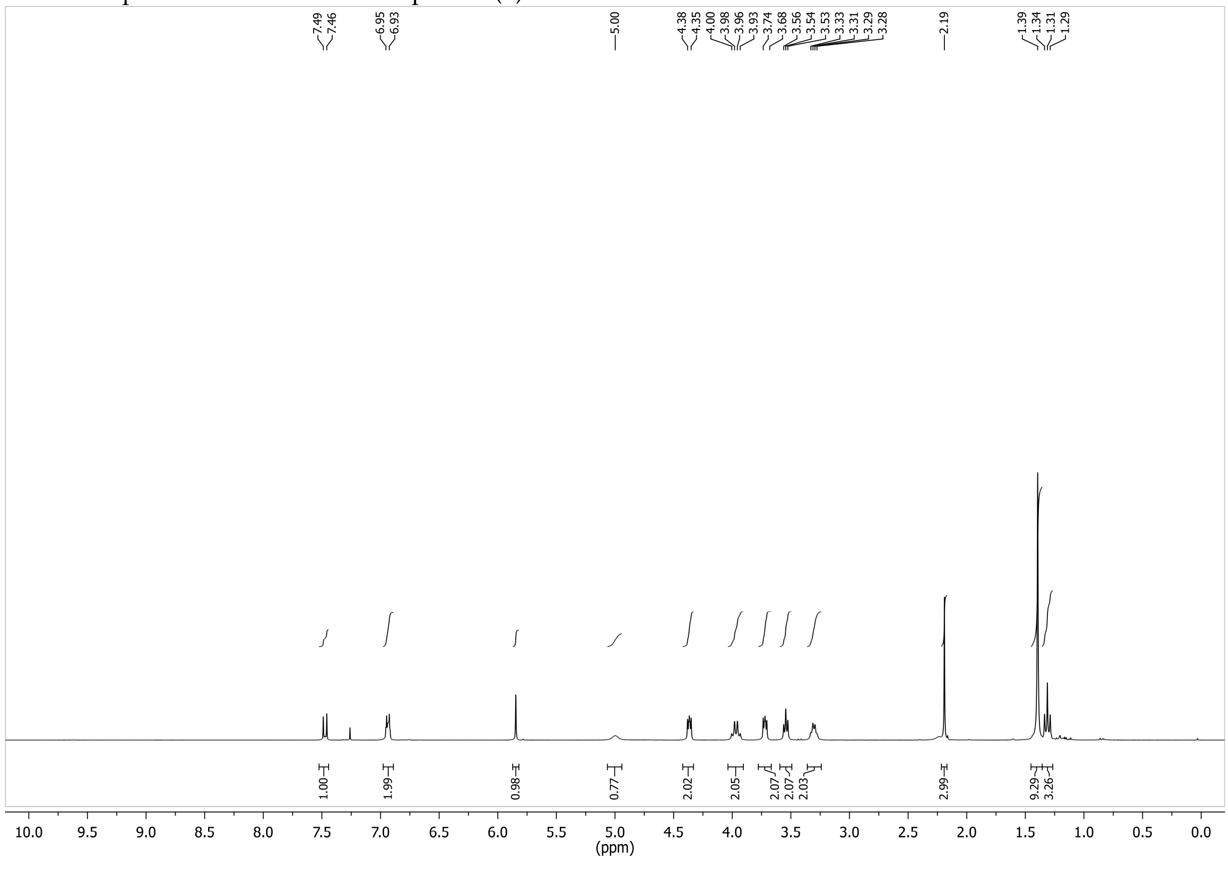

- DEPT-135 NMR spectrum in $CDCl_3$ of compound (**7**)

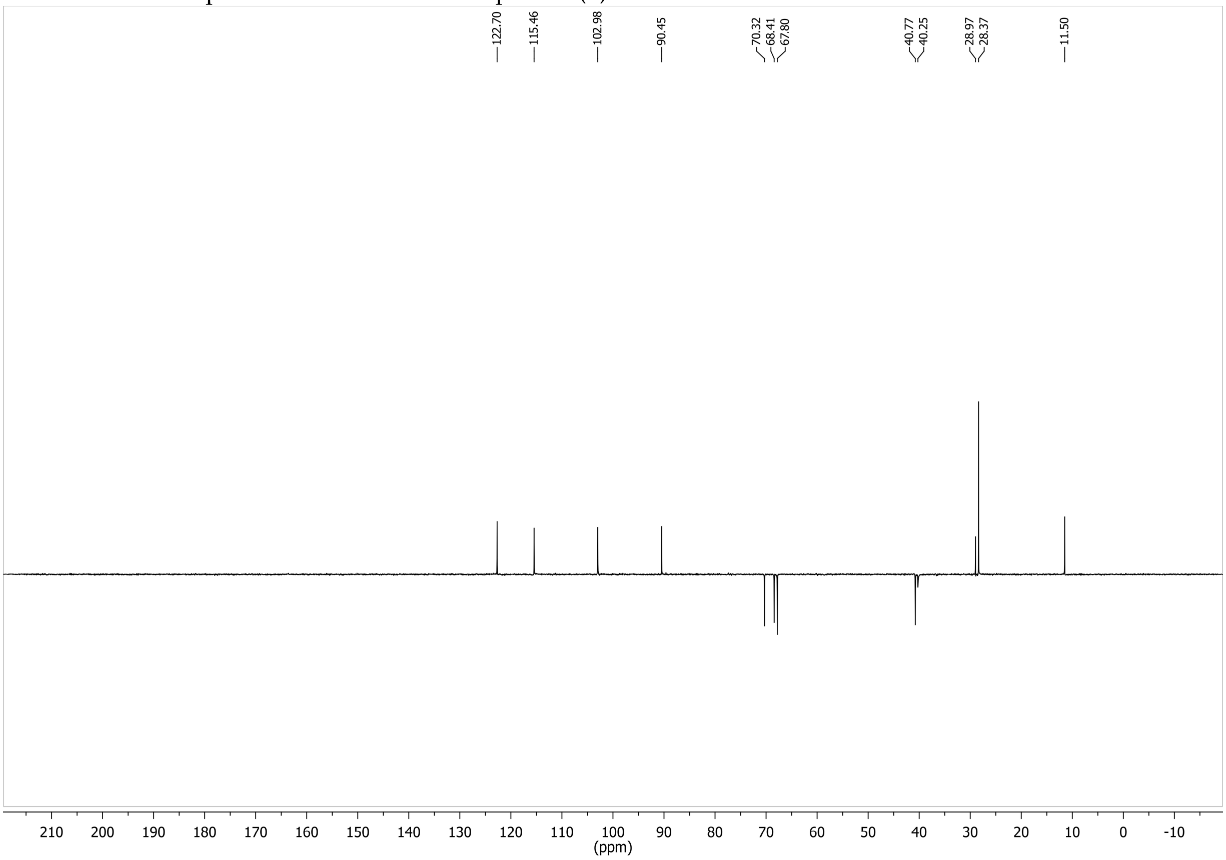

- ^{13}C NMR NMR spectrum in $CDCl_3$ of compound (**7**)

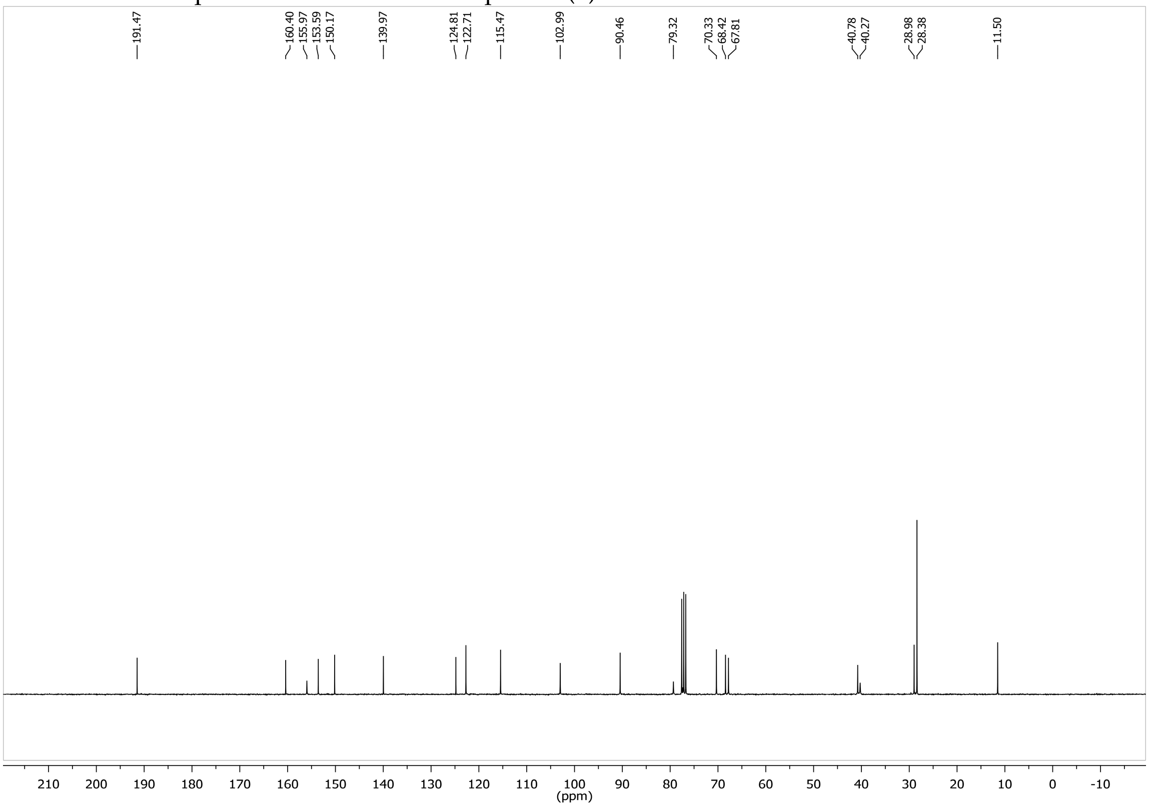

- ^{1}H NMR spectrum in DMSO-d6 of compound (**8**)

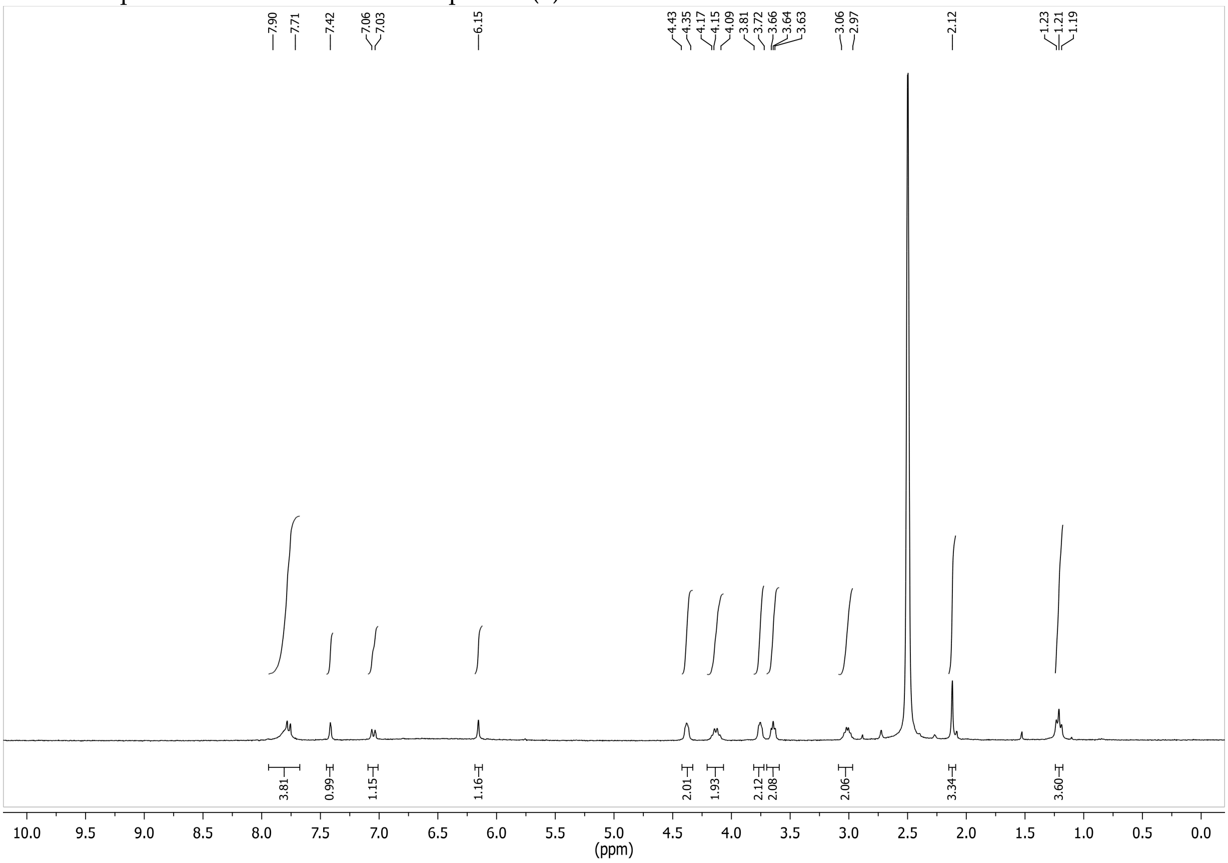

- DEPT-135 NMR spectrum in DMSO-d6 of compound (**8**)

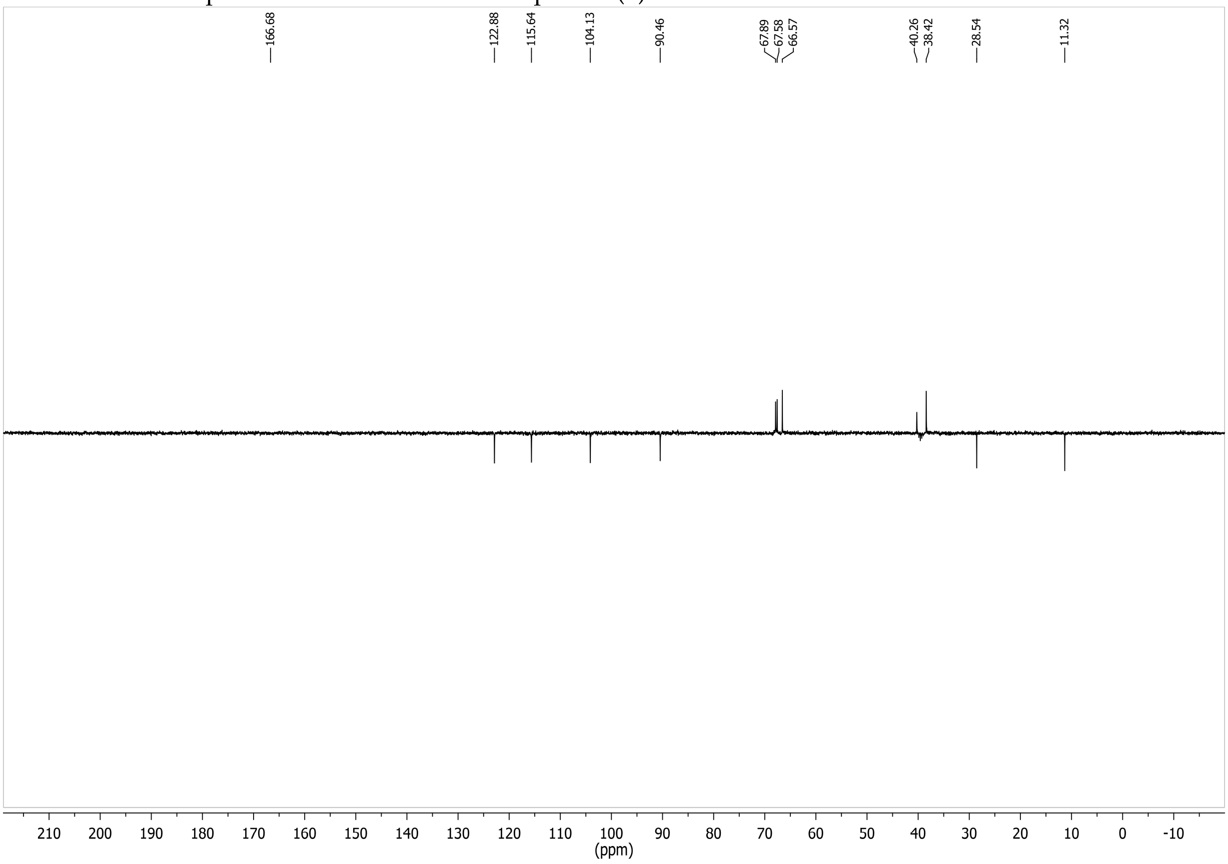

• ^{13}C NMR NMR spectrum in DMSO-d6 of compound (**8**)

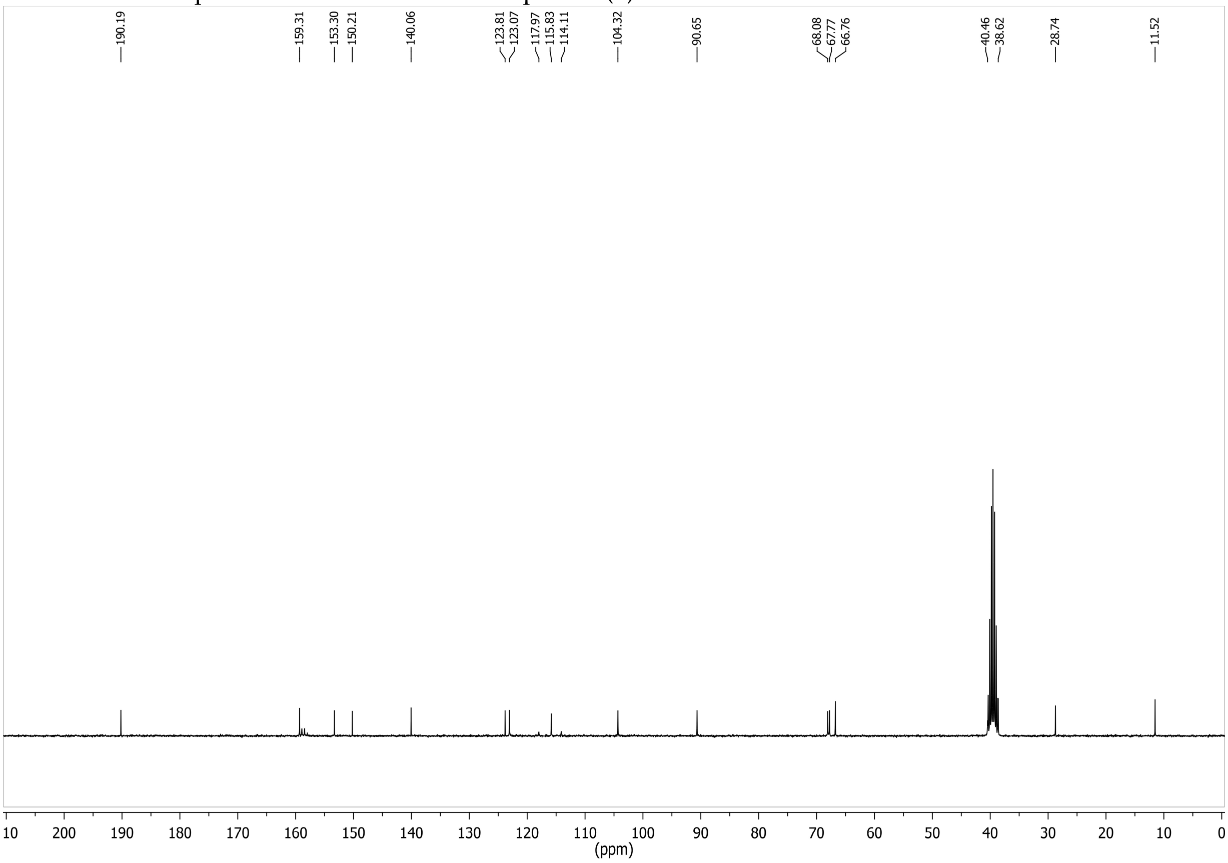

• ^{1}H NMR spectrum in CDCl3 of compound (**12**)

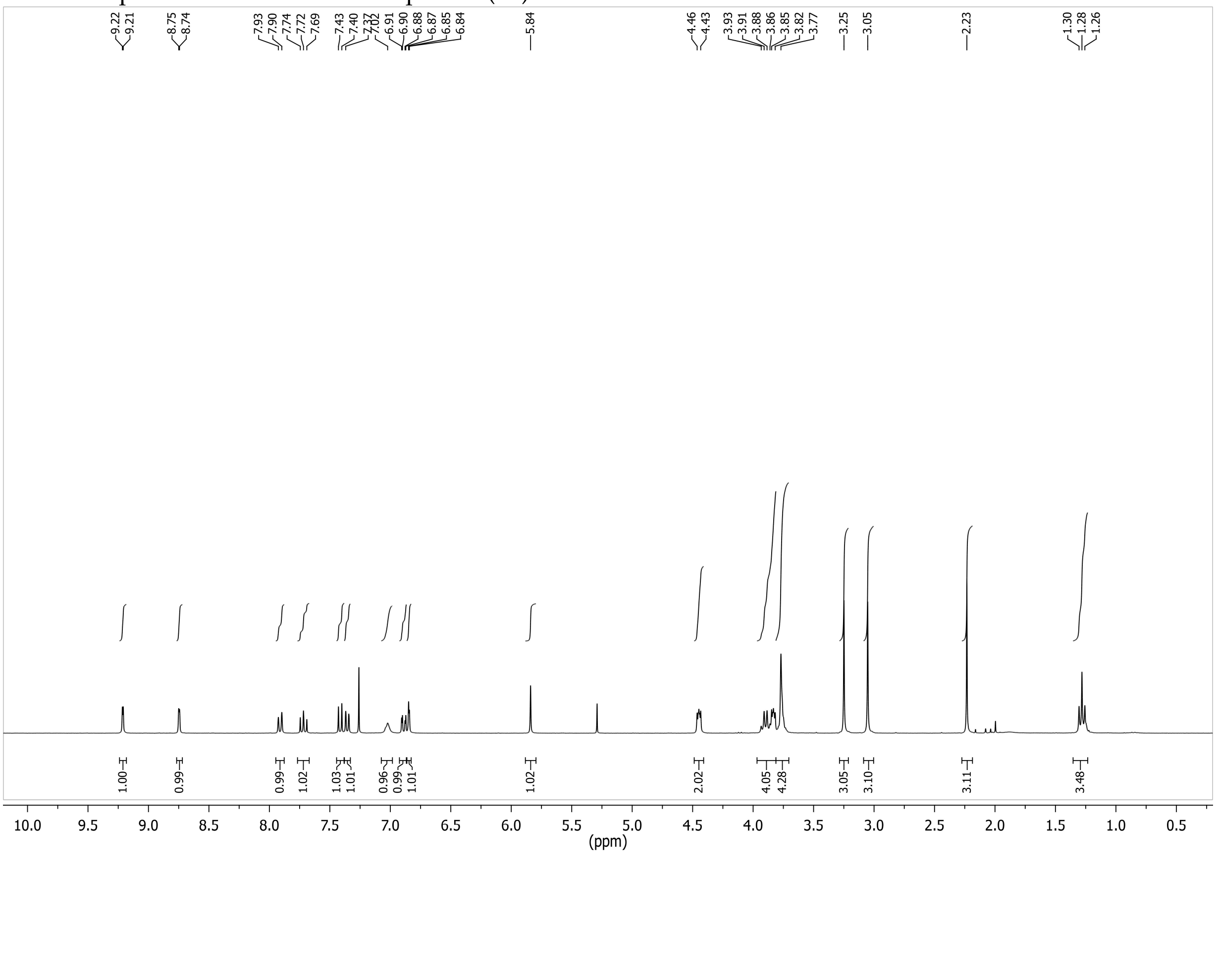

- DEPT-135 NMR spectrum in $CDCl_3$ of compound (**12**)

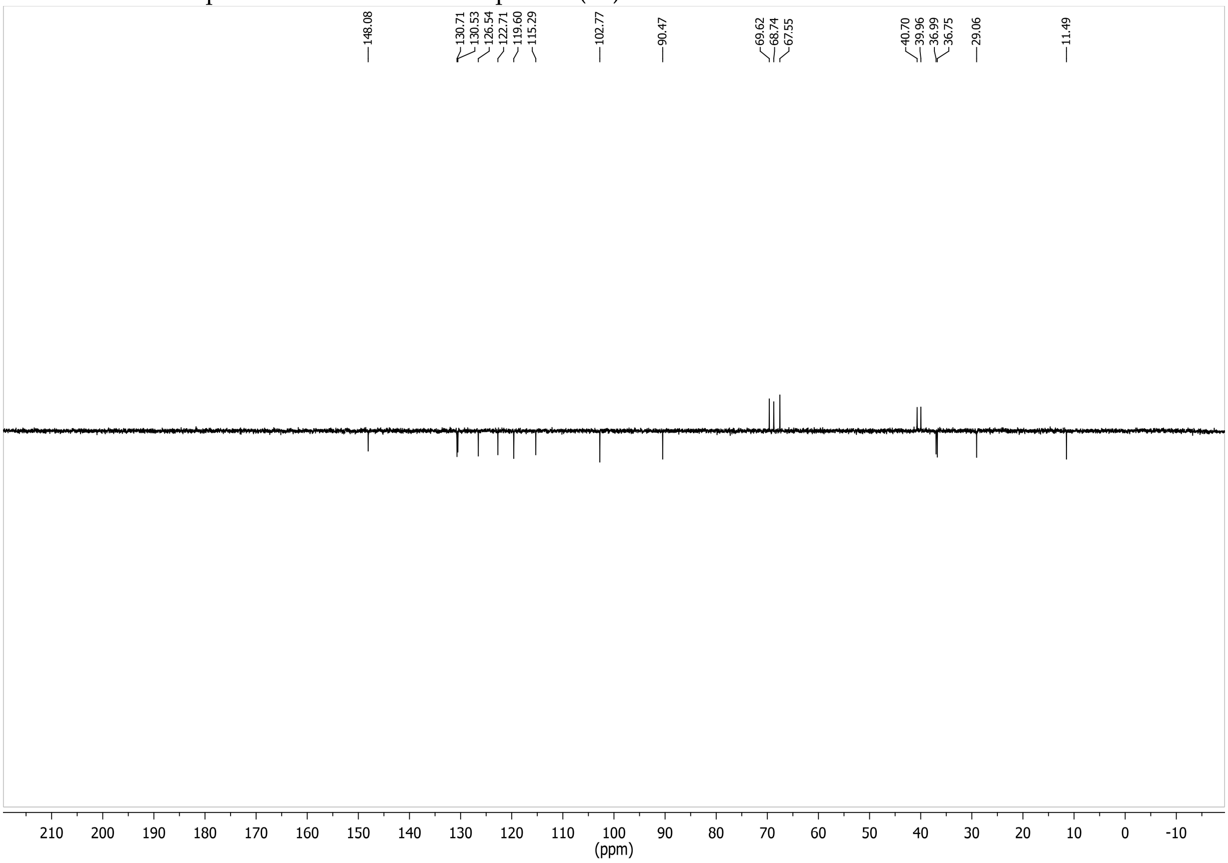

- ^{13}C NMR NMR spectrum in $CDCl_3$ of compound (**12**)

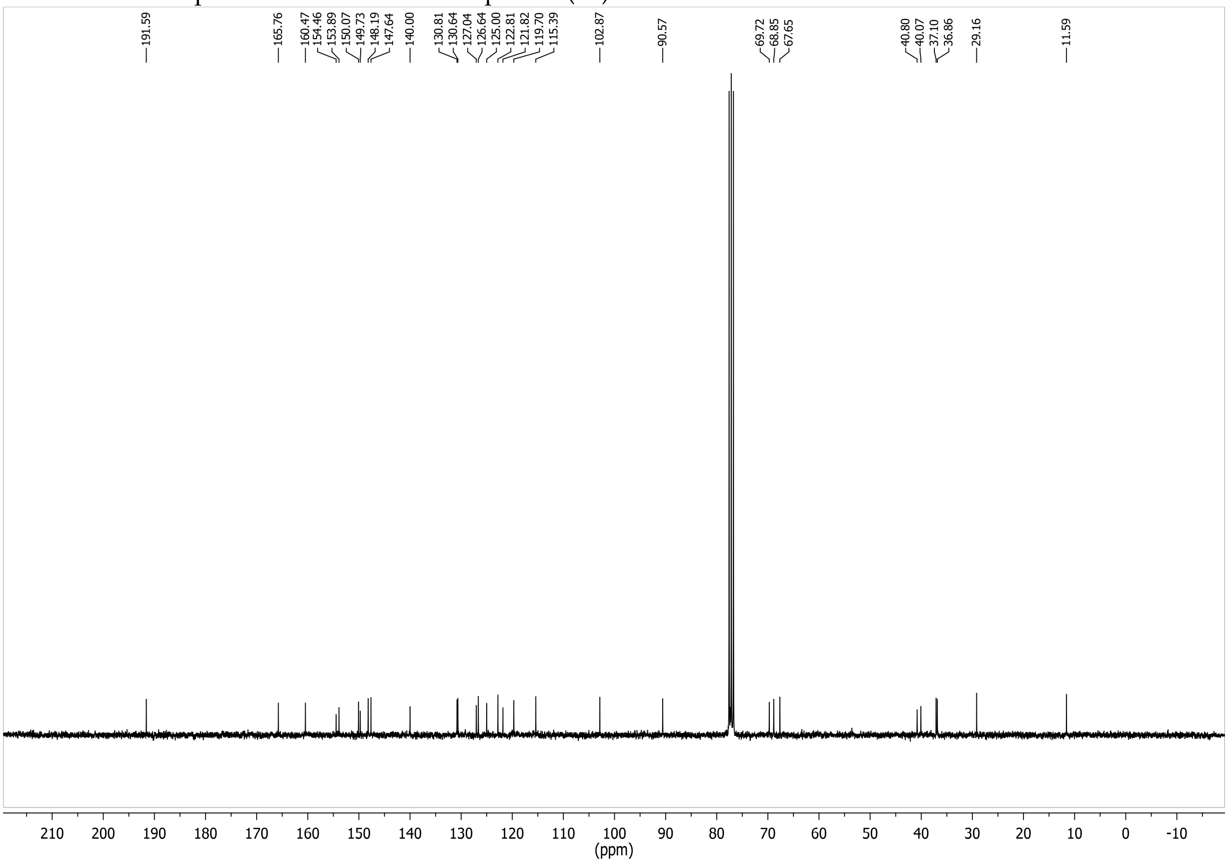

- ^1H NMR spectrum in $CDCl_3$ of compound (**13**)

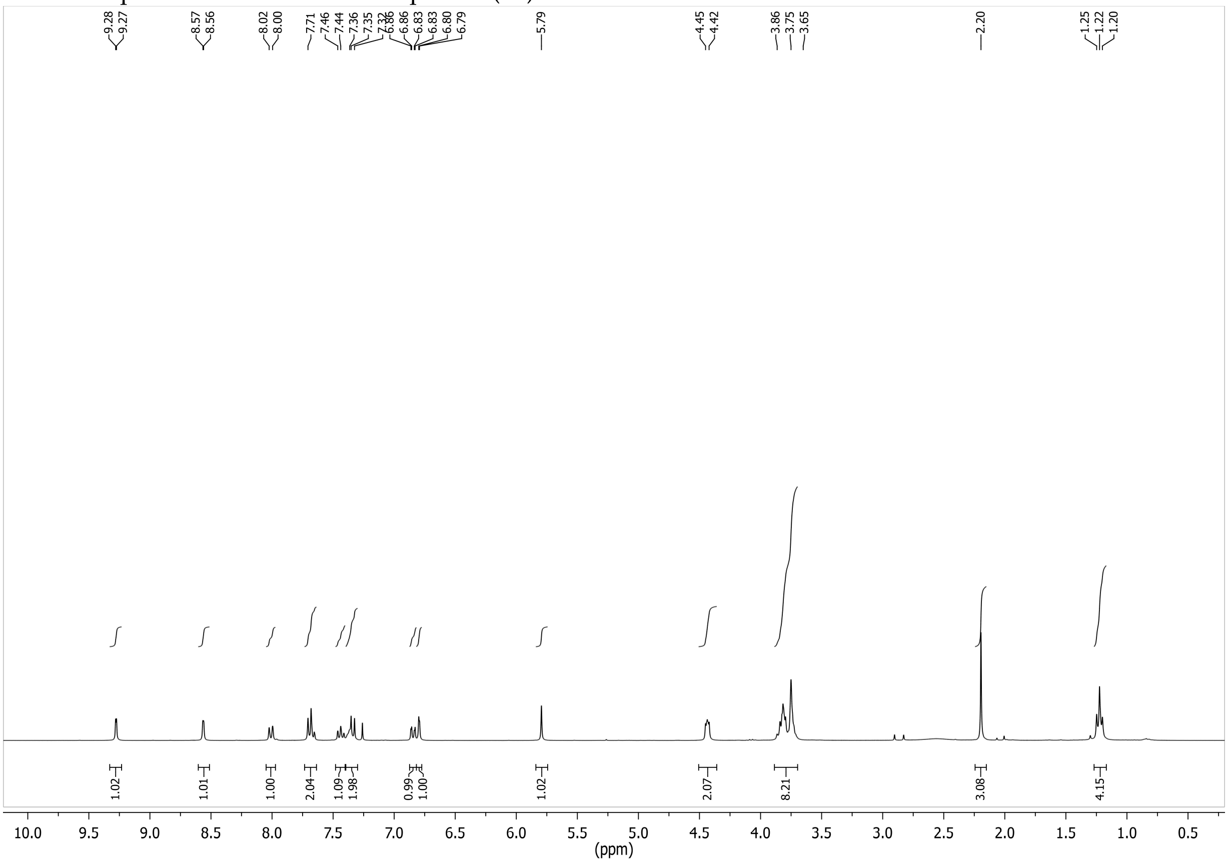

- DEPT-135 NMR spectrum in $CDCl_3$ of compound (**13**)

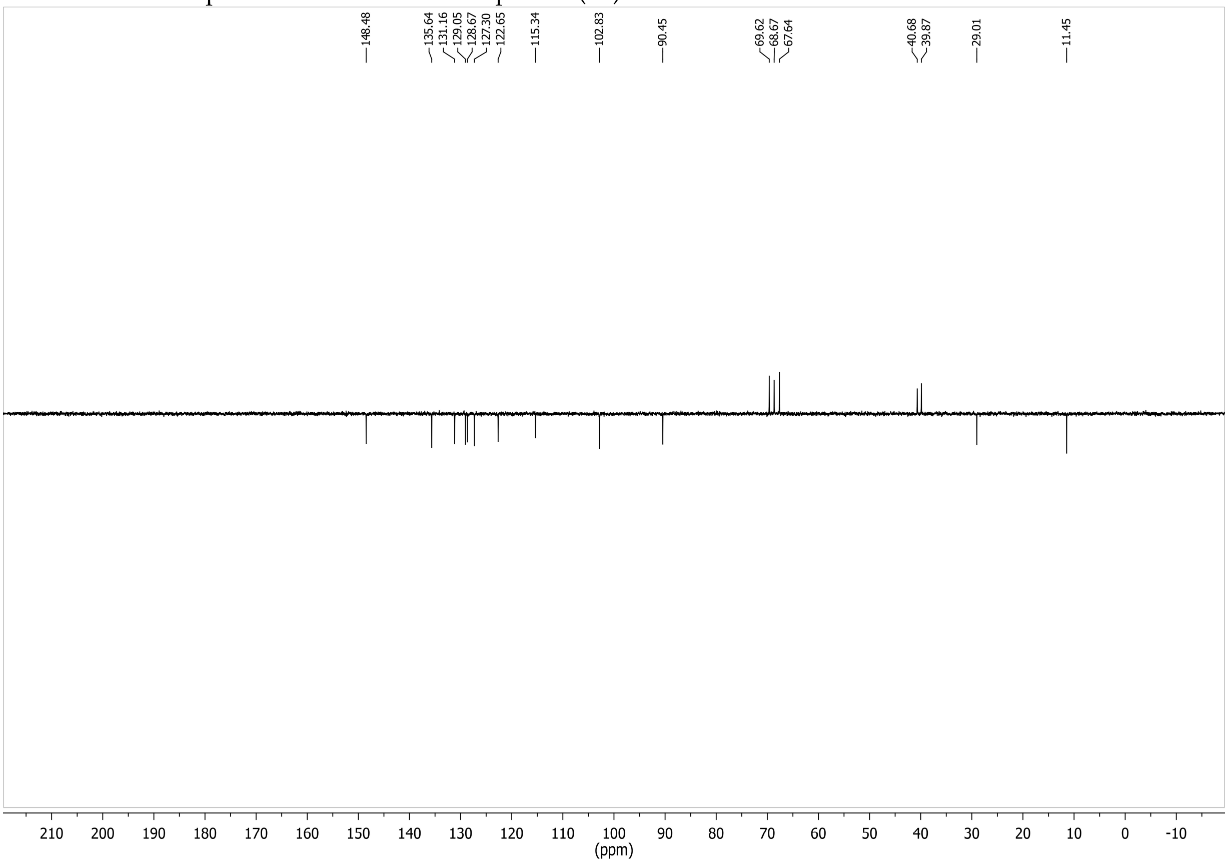

- ^{13}C NMR NMR spectrum in $CDCl_3$ of compound (**13**)

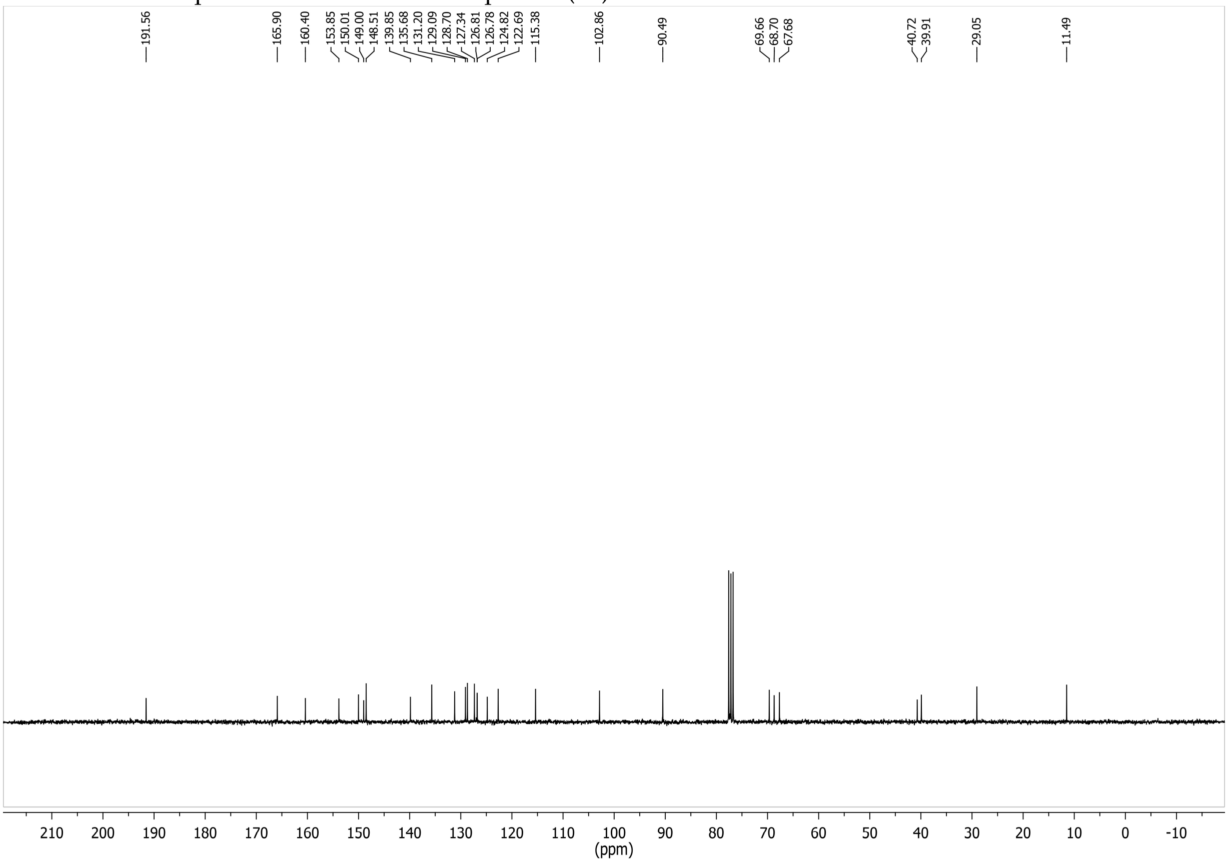

- ^{1}H NMR spectrum in DMSO-d_6 of compound (**14**)

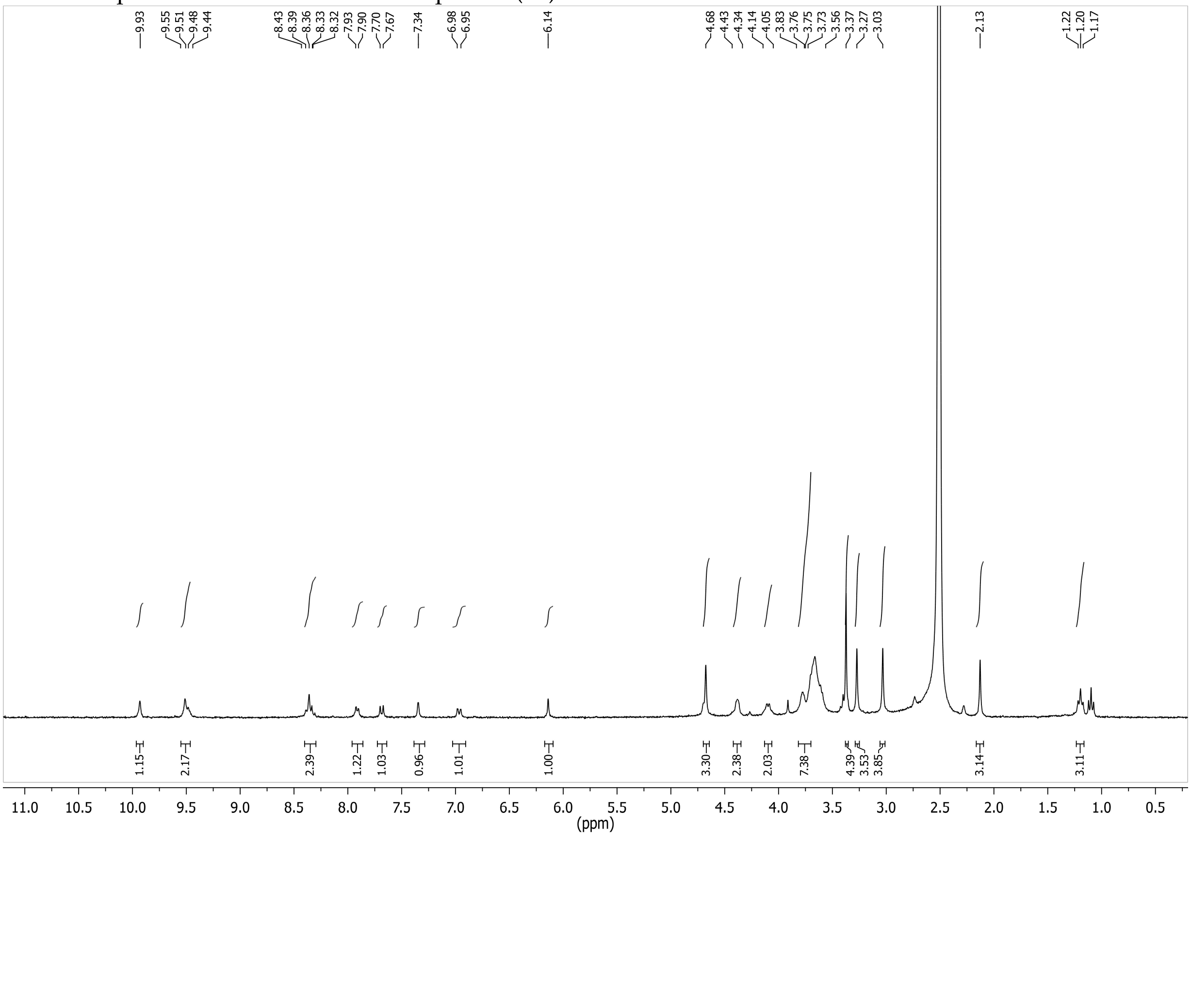

- DEPT-135 NMR spectrum in DMSO-d$_6$ of compound (**14**)

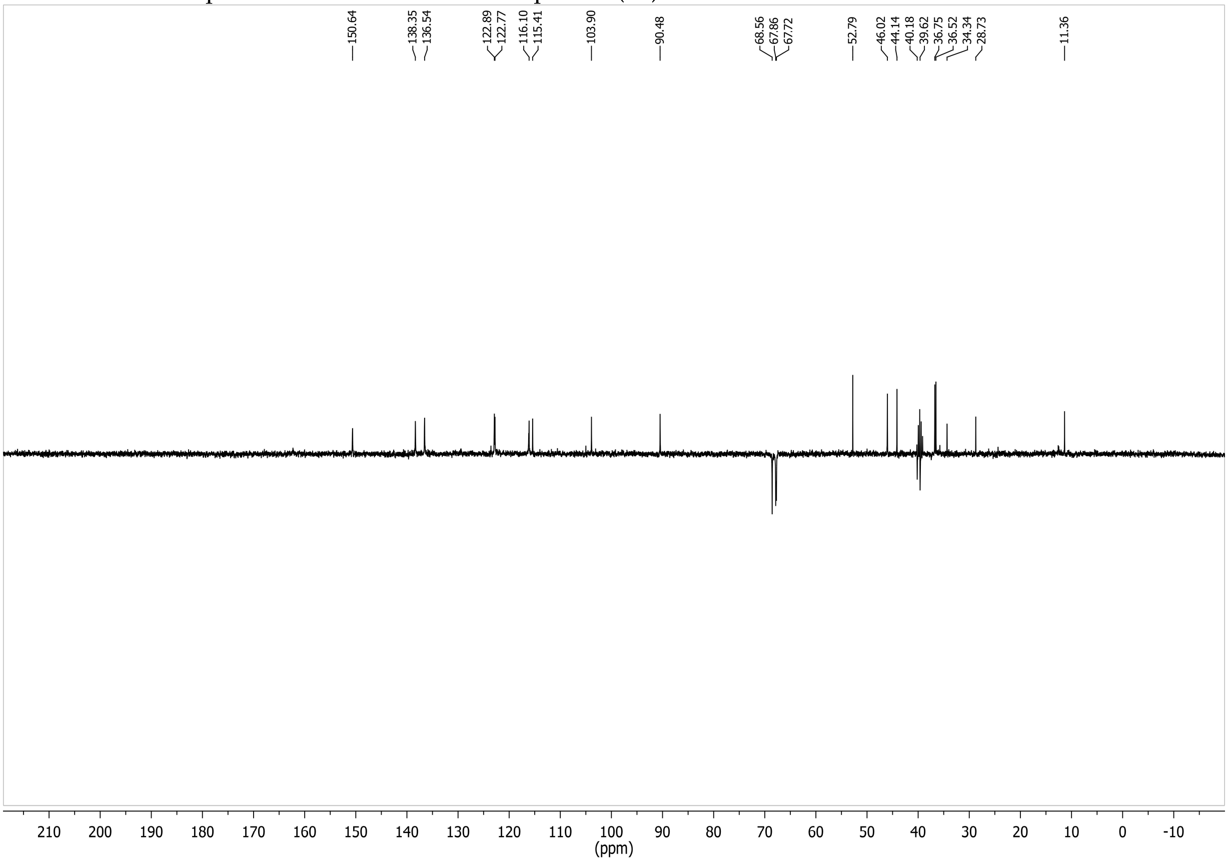

- ^{13}C NMR NMR spectrum in DMSO-d$_6$ of compound (**14**)

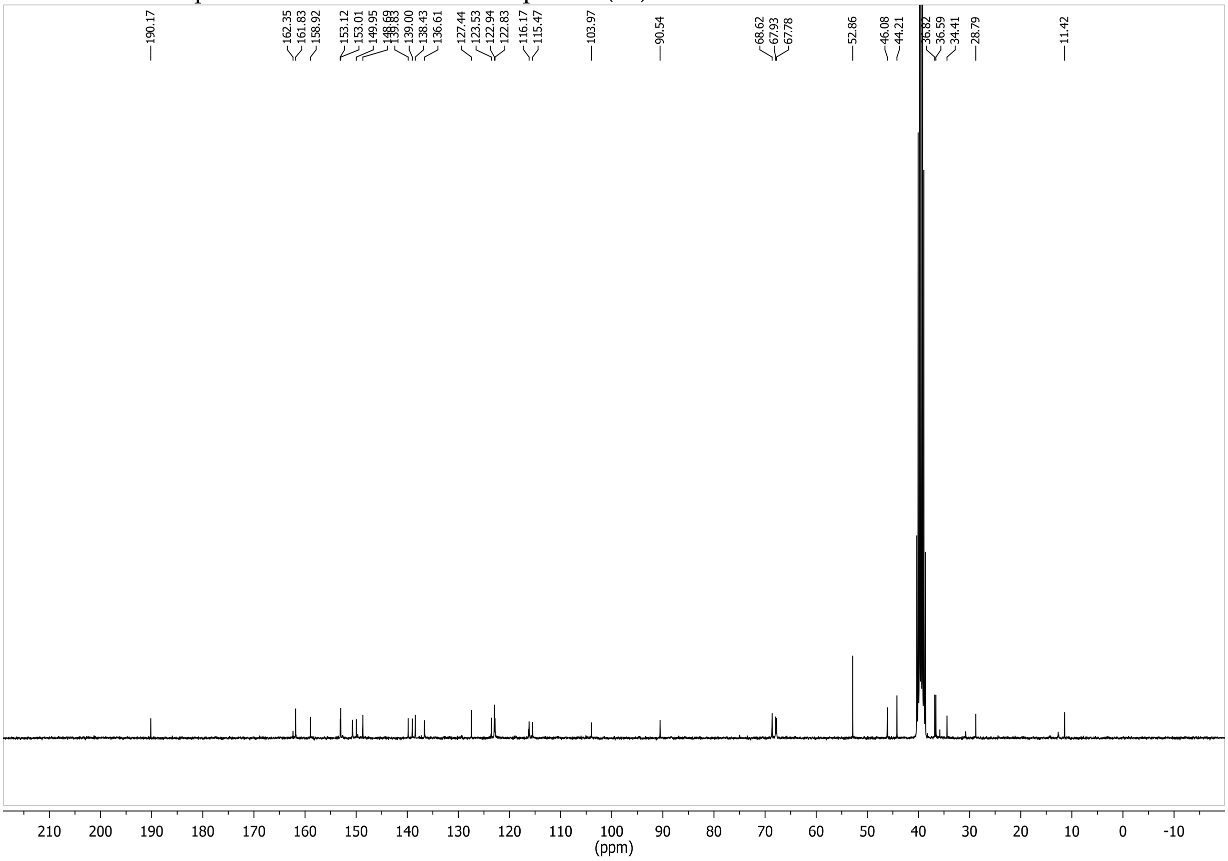

- ^{1}H NMR spectrum in DMSO-d_6 of compound (**4**)

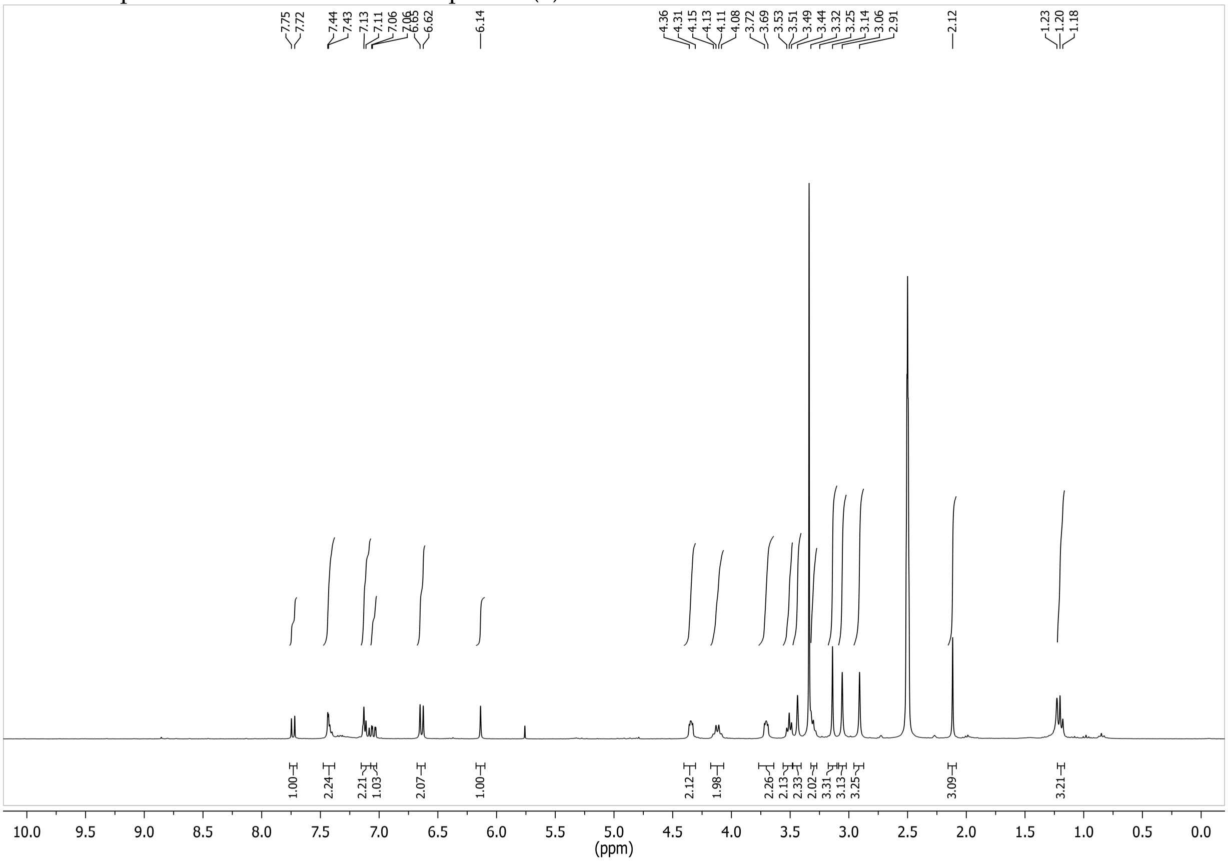

- DEPT-135 NMR spectrum in DMSO-d_6 of compound (**4**)

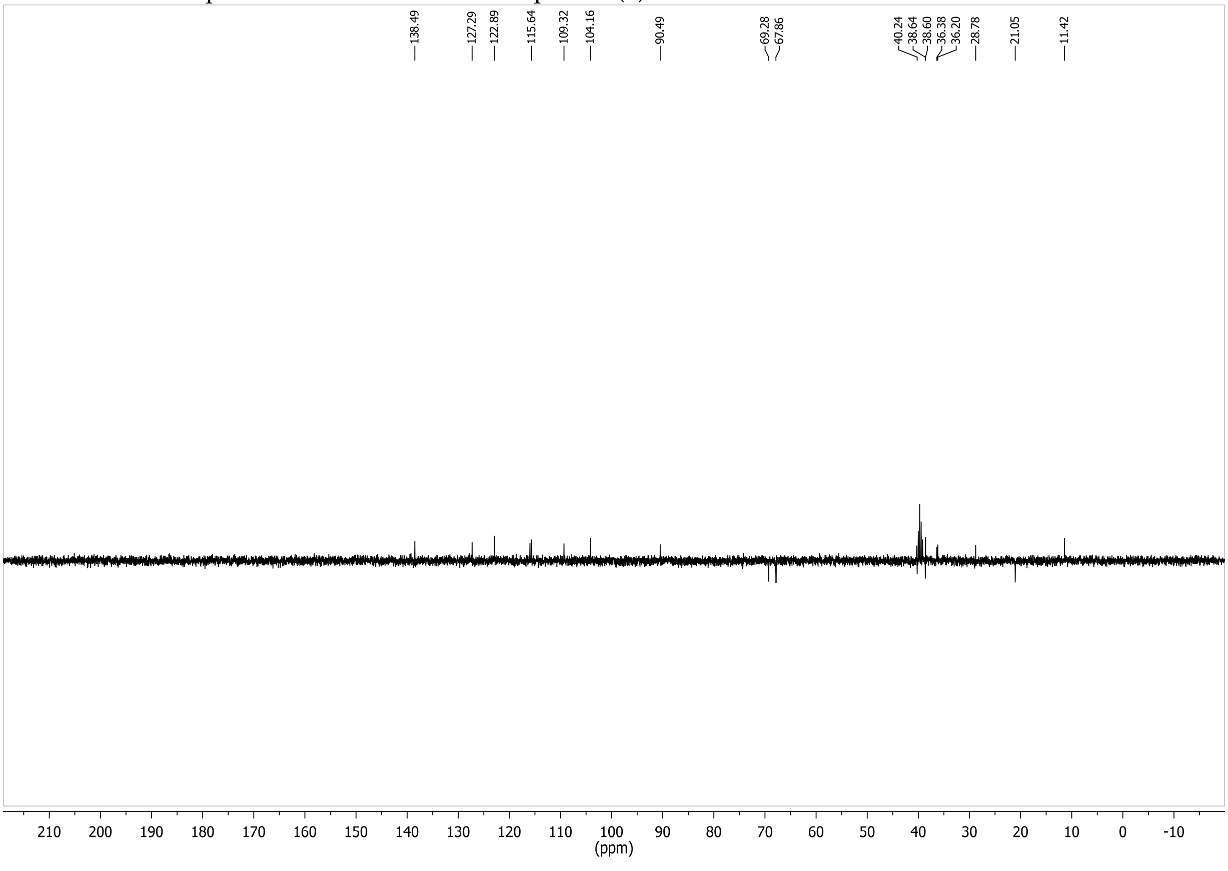

- ^{13}C NMR NMR spectrum in DMSO-d_6 of compound (**4**)

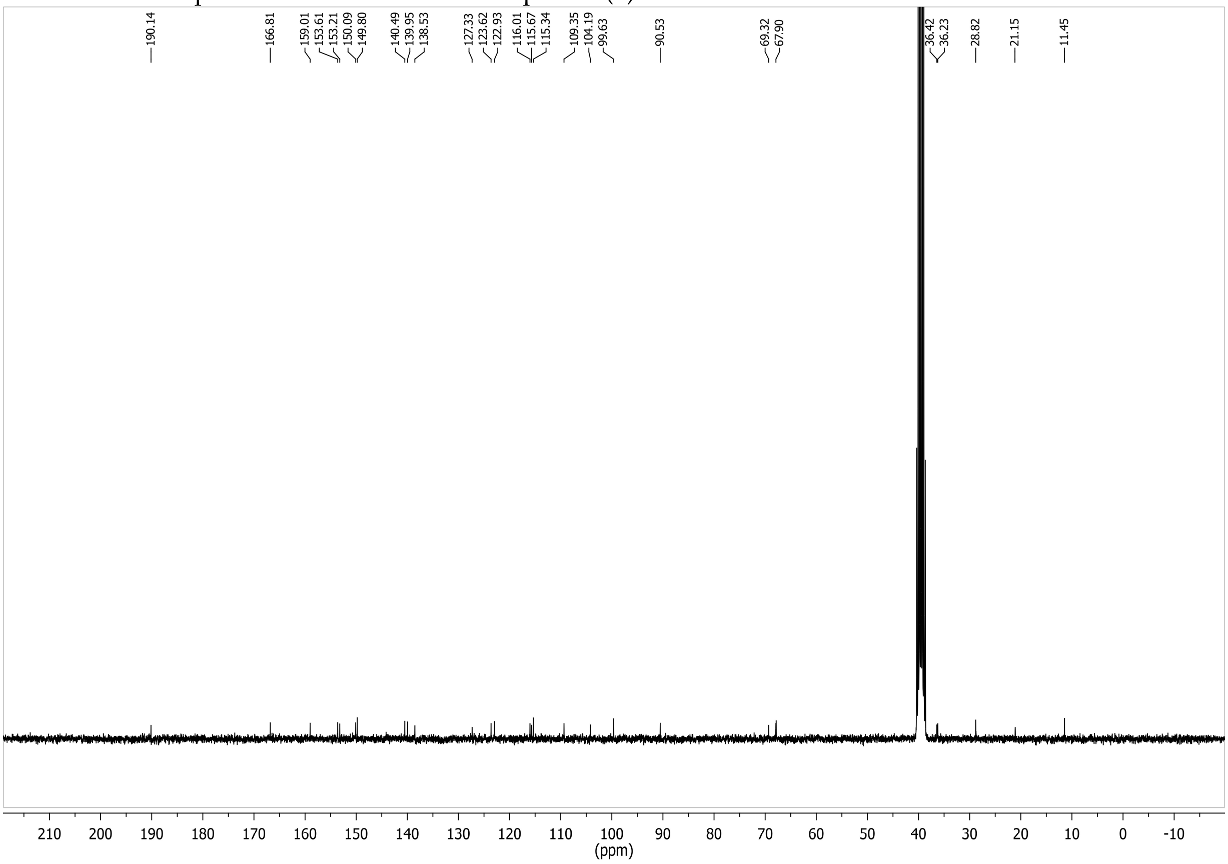

- ^{1}H NMR spectrum in DMSO-d_6 of compound (**15**)

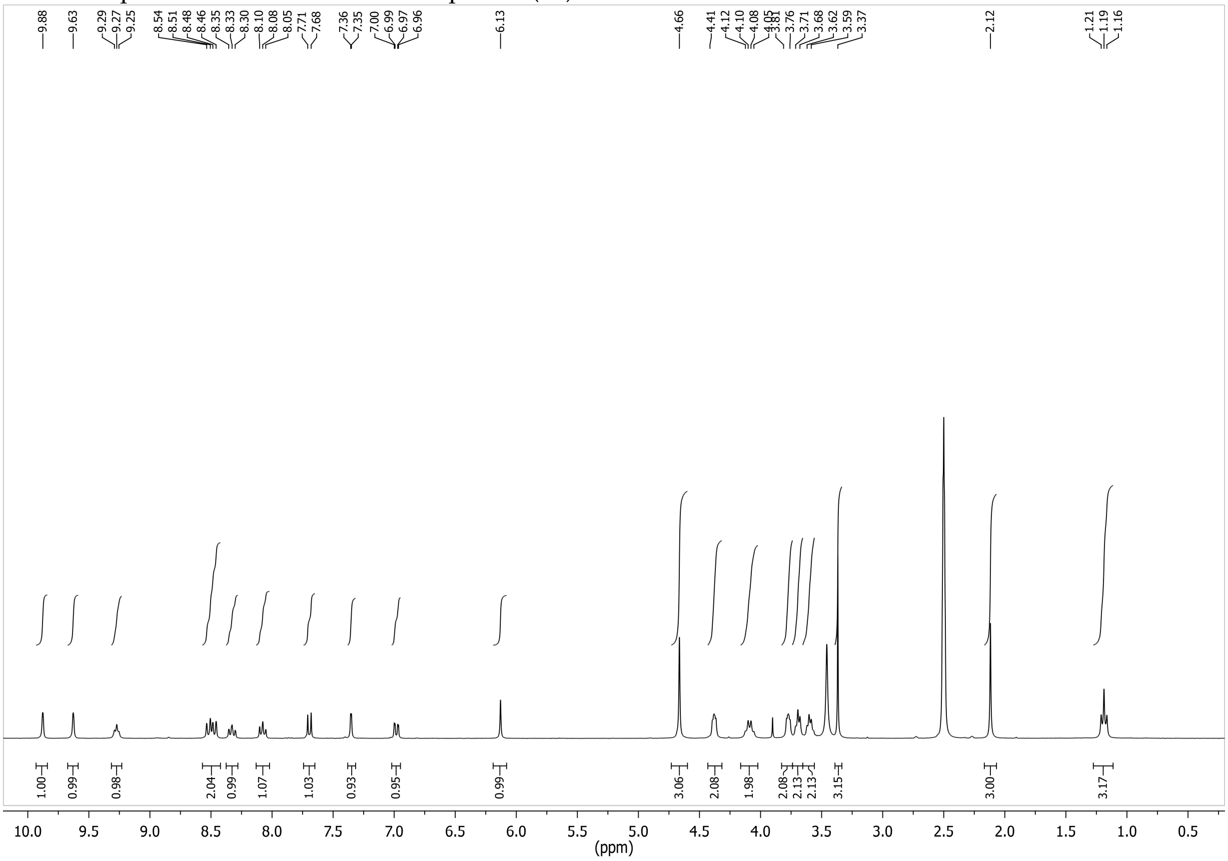

- DEPT-135 NMR spectrum in DMSO-d6 of compound (**15**)

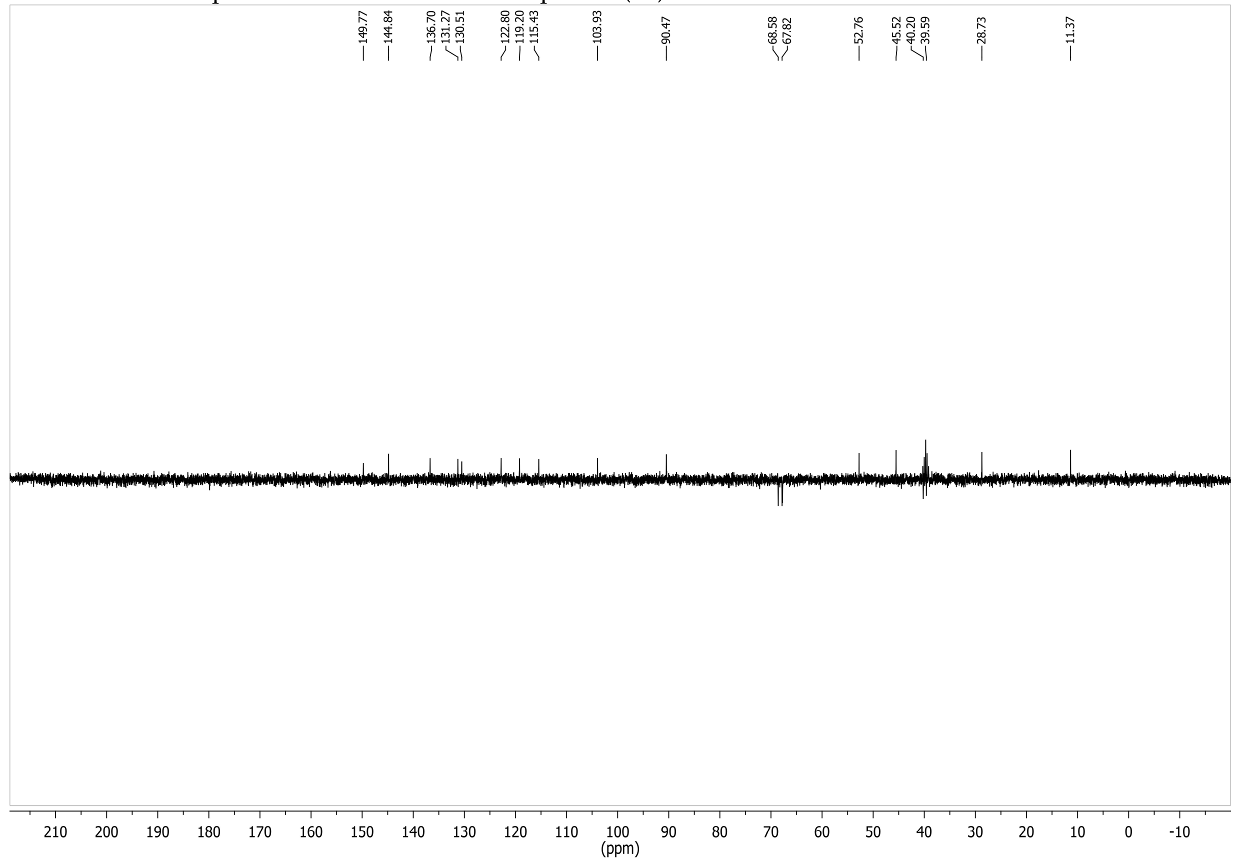

- ^{13}C NMR NMR spectrum in DMSO-d6 of compound (**15**)

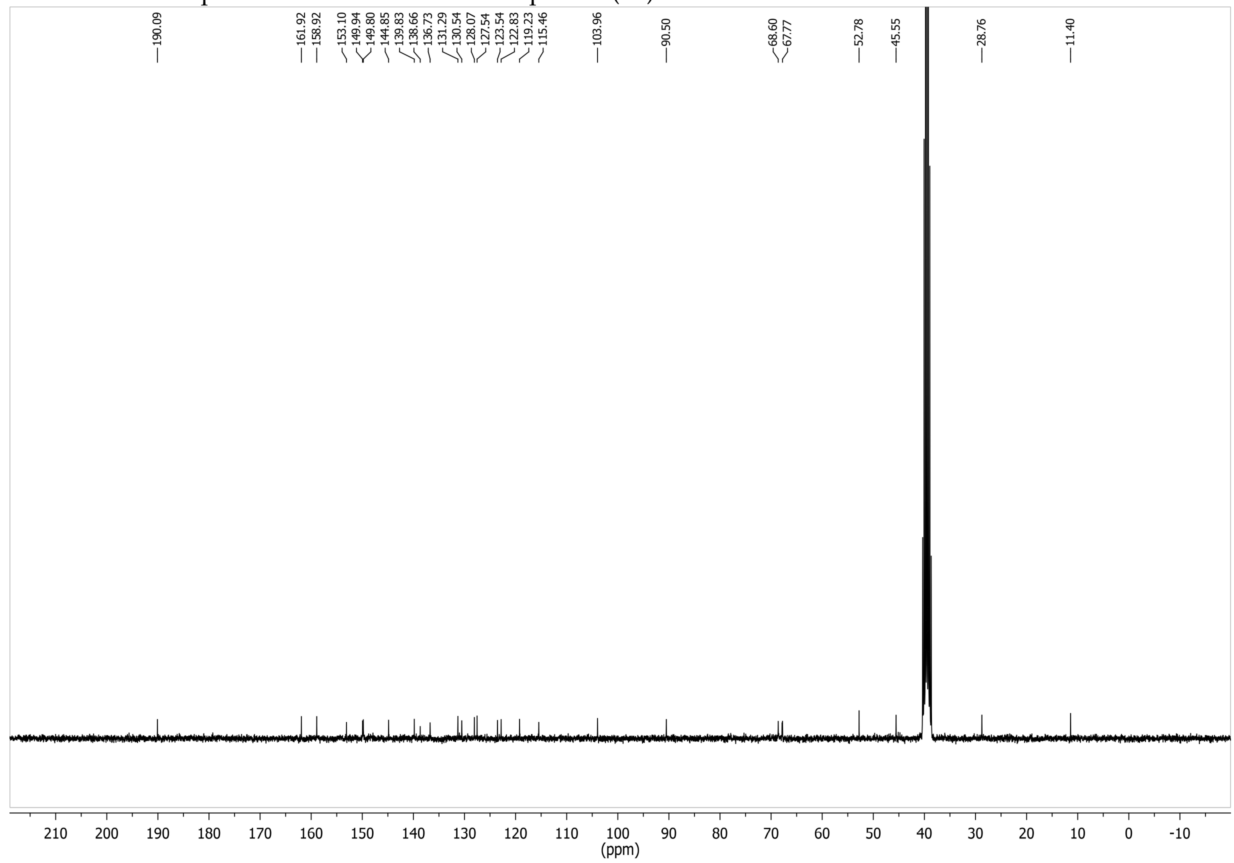

- ^1H NMR spectrum in $CDCl_3$ of compound (**16**)

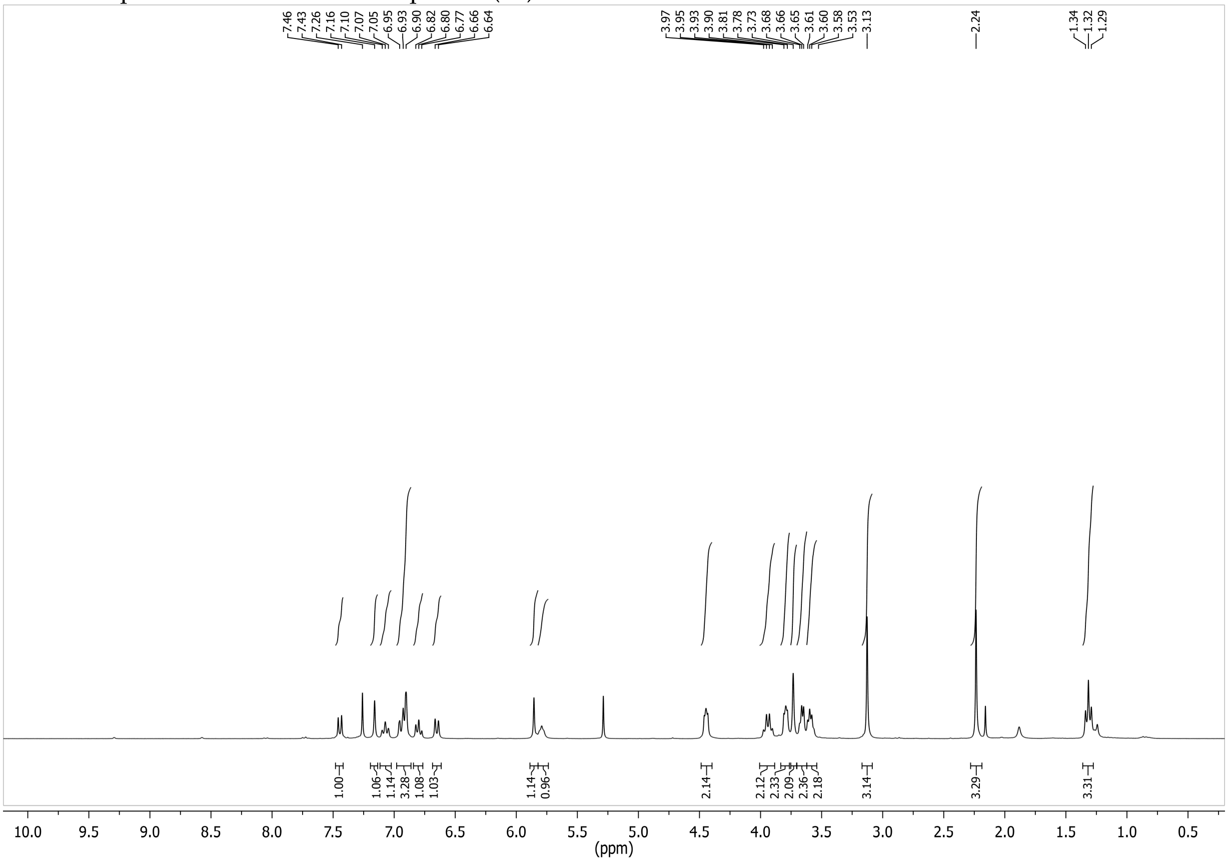

- DEPT-135 NMR spectrum in $CDCl_3$ of compound (**16**)

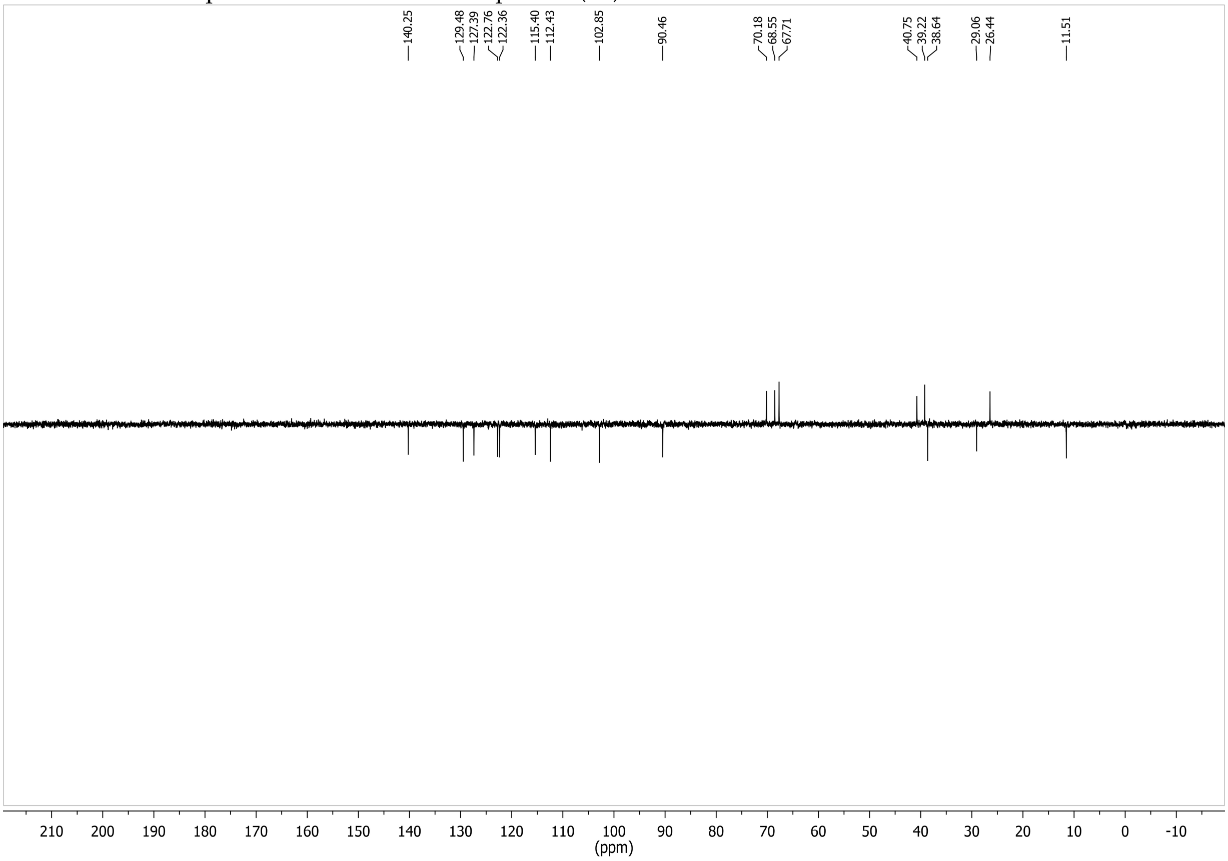

- ^{13}C NMR NMR spectrum in $CDCl_3$ of compound (**16**)

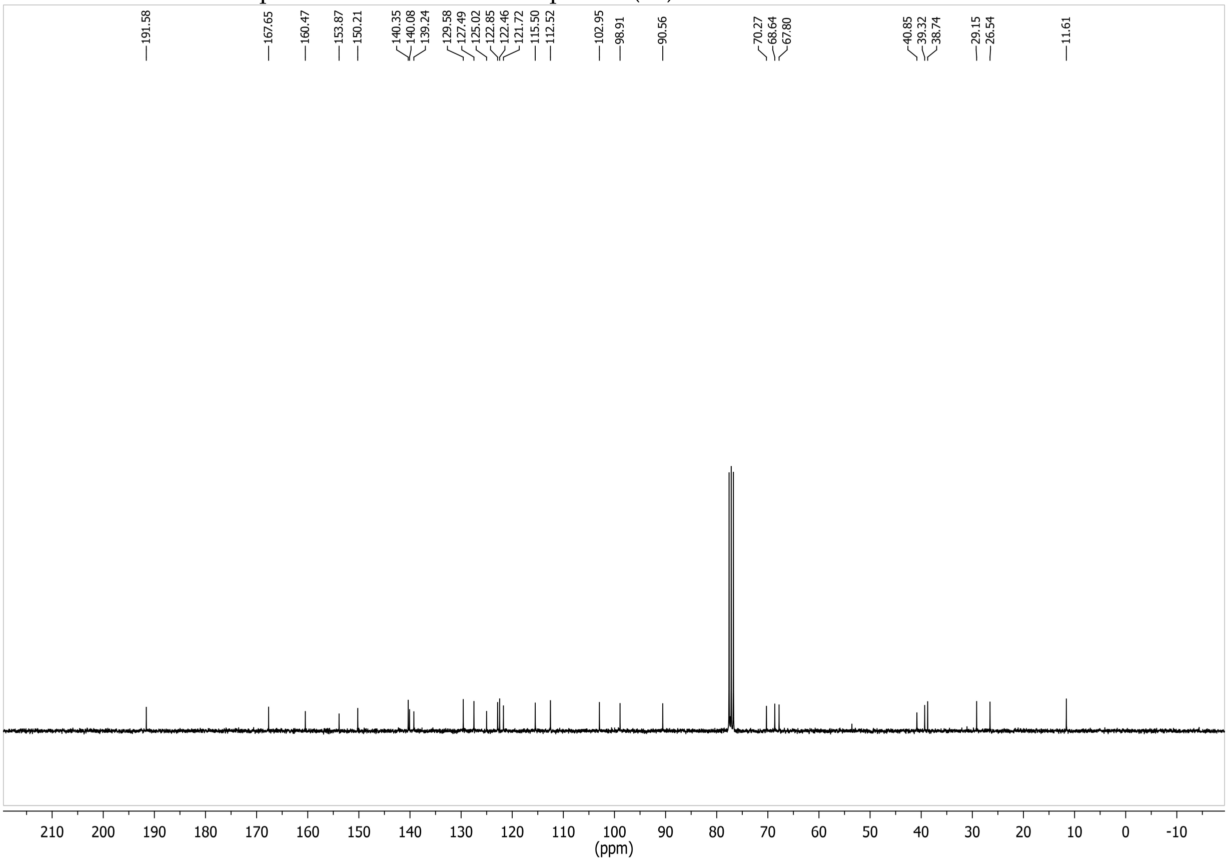

Supplement: Supplementary file 1 [file molecules-24-01264-s001.pdf]
